# Model Balancing: A Search for In-Vivo Kinetic Constants and Consistent Metabolic States

**DOI:** 10.3390/metabo11110749

**Published:** 2021-10-29

**Authors:** Wolfram Liebermeister, Elad Noor

**Affiliations:** 1 INRAE, MaIAGE, Université Paris-Saclay, 78350 Jouy-en-Josas, France; 2 Department of Plant and Environmental Sciences, Weizmann Institute of Science, Rehovot 7610001, Israel; elad.noor@weizmann.ac.il

**Keywords:** metabolic model, enzyme kinetic constant, parameter estimation, convex optimality problem, parameter balancing, enzyme cost minimisation

## Abstract

Enzyme kinetic constants in vivo are largely unknown, which limits the construction of large metabolic models. Given measured metabolic fluxes, metabolite concentrations, and enzyme concentrations, these constants may be inferred by model fitting, but the estimation problems are hard to solve if models are large. Here we show how consistent kinetic constants, metabolite concentrations, and enzyme concentrations can be determined from data if metabolic fluxes are known. The estimation method, called model balancing, can handle models with a wide range of rate laws and accounts for thermodynamic constraints between fluxes, kinetic constants, and metabolite concentrations. It can be used to estimate in-vivo kinetic constants, to complete and adjust available data, and to construct plausible metabolic states with predefined flux distributions. By omitting one term from the log posterior—a term for penalising low enzyme concentrations—we obtain a convex optimality problem with a unique local optimum. As a demonstrative case, we balance a model of *E. coli* central metabolism with artificial or experimental data and obtain a physically and biologically plausible parameterisation of reaction kinetics in *E. coli* central metabolism. The example shows what information about kinetic constants can be obtained from omics data and reveals practical limits to estimating in-vivo kinetic constants. While noise-free omics data allow for a reasonable reconstruction of in-vivo $k_{\mathrm{cat}}$ and $K_{M}$ values, prediction from noisy omics data are worse. Hence, adjusting kinetic constants and omics data to obtain consistent metabolic models is the main application of model balancing.

## 1. Introduction

As the number of reconstructed metabolic networks is growing, so is the desire to populate these networks with enzymatic rate laws inferred from omics data [1,2,3,4,5]. To convert networks into kinetic models, one needs to determine arrows for small-molecule regulation, choose enzymatic rate laws, determine the kinetic constants, and construct plausible metabolic states (characterised by enzyme concentrations, metabolite concentrations, and fluxes). Over the years, different approaches have been suggested to address these issues. For example, Liebermeister et al. proposed standardised rate laws [1,6] to replace unknown rate laws and to automate model construction. These rate laws were used to automatically build models [7] and evaluate them for their practical use [8]. It is common to populate models with in-vitro kinetic constants [9,10]. Although online databases such as BRENDA [11,12] collect and aggregate many of these measured parameters, large gaps remain when dealing with larger models or relatively unstudied organisms. An alternative, suggested by Heckmann et al. [13], is to infer unknown $k_{\mathrm{cat}}$ values by machine learning. Alternatively, one can estimate in-vivo kinetic constants directly from flux [14], metabolite, and enzyme data [15,16], or use the kinetic model itself to fit its parameters to collected data [17,18,19]. On the flip side, too much data on kinetic parameters can also pose a problem. For instance, Wegscheider and Haldane conditions (which are derived directly from the laws of thermodynamics) impose strict dependencies between equilibrium and kinetic constants and are likely to be violated when all the parameters are measured independently [1]. Methods for constructing thermodynamically consistent parameter sets have been proposed [20,21,22,23,24]. For instance, Stanford et al. [5] applied these methods for genome-scale metabolic network. Currently, one can easily find large kinetic models [2,25] as well as workflows for model parameterisation [26,27]. Moreover, parameter sampling and ensemble modelling [28,29,30,31,32] can yield plausible parameter sets [33] and help us reach conclusions about dynamic behaviour independently of specific parameter values [34].

How can omics data be used to infer in-vivo kinetic constants? Methods for parameterising metabolic models use different types of knowledge (in-vitro kinetic constants, omics data, and physical parameter constraints) and different estimation approaches (including machine learning, regression models, calculations based on rate laws, and model fitting).

*1. Estimating $k_{\mathrm{cat}}$ values by taking the maximum over apparent catalytic rates.* To estimate $k_{\mathrm{cat}}$ values, Davidi et al. [15] combined proteomics data with flux data from flux balance analysis (FBA). The method, which was later called kinetic profiling [35], assumes unknown rate laws v=ek(c) with flux *v*, enzyme concentration *e*, and a catalytic rate *k* depending on unknown metabolite concentrations (vector c). To determine $k_{\mathrm{cat}}$ from data, the method computes the apparent rate $k_{\mathrm{app}}^{s}=v^{s}/e^{s}$ in a number of metabolic states *s*. Since $k_{\mathrm{cat}}$ is the maximal catalytic rate of an enzyme (i.e., $k_{\mathrm{cat}}\geq k\left(c\right)$), the maximum value of $k_{\mathrm{app}}^{s}$ across the different states satisfies the same inequality, namely $k_{\mathrm{cat}}\geq \max_{s}k_{\mathrm{app}}^{s}$. If each enzyme reaches its maximal capacity in one of the states, then the maximum value becomes a good estimator for $k_{\mathrm{cat}}$. Applied to *E. coli* data, this method was able to provide relatively precise estimates of $k_{\mathrm{cat}}$ values when compared to literature data (from the BRENDA database). The simplicity of the method makes it scalable and easily applicable to any organism whose metabolic network has been reconstructed. However, there are a few severe limitations. First, due to the max function, a single large outlier value can completely distort the result. Such outliers may arise if measured protein concentrations are small and imprecise. Another caveat is that an enzyme may stay below its maximal capacity in all of the samples, especially if the number of measured metabolic states is small or when considering complex (compartmentalised) cells. This could explain why the correlation between $\max_{s}k_{\mathrm{app}}^{s}$ and literature $k_{\mathrm{cat}}$ values was lower for plants than for *E. coli* [36].

*2. Ensemble modelling.* If model parameters or state variables are sampled from random distributions, we obtain a model ensemble. Ensemble modelling can be used to estimate the likelihood of different states of a system, in line with our limited data and knowledge. In Structural Kinetic Modelling (SKM) [34], model parameters are chosen randomly to create a model ensemble. First, a metabolic state is defined by choosing fluxes and metabolite concentrations. Then, rate laws are chosen in agreement with this state by randomly sampling the saturation values of enzymes and reconstructing the corresponding kinetic constants. Structural Thermokinetic Modelling (STM) [37], a variant of SKM, considers reversible rate laws and implements thermodynamic constraints. SKM and STM can use priors or data for $K_{M}$ values, but not for $k_{\mathrm{cat}}$ values and enzyme concentrations, and there is no simple way of fitting kinetic constants simultaneously to more than one metabolic state.

*3. Parameter estimation.* Parameter estimation can be seen as ensemble modelling in reverse. While ensemble modelling relies on a given distribution of model parameters for forward prediction, parameter estimation establishes such distributions from data, and possibly from prior information about the model (network structure) and its parameters (prior distributions). In practice, parameter fitting can be performed by optimisation or Monte Carlo methods such as random screening, genetic algorithms, or simulated annealing. For example, one may generate a large ensemble of possible parameter sets, compute the likelihood values for each of them, and choose the best parameter set (see [33] for an example). Optimisation by sampling is generic and easy to implement, but the larger the parameter spaces are, the more important it becomes to understand the optimality problem, and whether local optima can be expected. Knowing whether a problem is convex makes optimisation tasks more transparent.

*4. Fitting kinetic constants in single reactions.* If the flux, enzyme concentration, and metabolite concentrations in a reaction are known for several states, the kinetic constants can be fitted [38], for example by the SIMMER method [16]. Treating reactions one by one is much easier than fitting all constants for an entire network simultaneously. However, this approach has some limitations: complete information is typically unavailable for most reactions. Moreover, kinetic constants that were estimated independently for different reactions may violate Wegscheider conditions.

Despite all these efforts, the problem of inferring in-vivo kinetic constants is largely unsolved: the $k_{\mathrm{cat}}$ values in-vivo are hard to measure, and in-vitro values can be unreliable. If our aim is to obtain complete, consistent kinetic constants for a given kinetic model, two distinct approaches can be taken: (i) starting from known in-vitro kinetic constants and adjusting and completing them based on omics data, to obtain a consistent, fully parameterised kinetic model, or (ii) estimating in-vivo kinetic constants directly from omics data. In both cases, we consider a kinetic model and fit its parameters (i.e., $K_{M},k_{\mathrm{cat}}$, etc.). Ideally, if enzyme concentrations, metabolite concentrations and fluxes were known precisely for a sufficient number of metabolic states, we could directly compute kinetic constants [16] that satisfy the kinetic rate laws in all metabolic states. Conversely, if kinetic constants and enzyme concentrations were known precisely, metabolite concentrations and fluxes could be computed by simulating the model. Of course, neither one of these ideals is realistic: typically, data are incomplete and imprecise and cannot be directly inserted into the models. For a complete picture and reliable estimates, these heterogeneous data must be combined—but how? In practice, this depends on our aims: on the one hand, we may use an existing model for checking data consistency or augmenting it, e.g., by estimating in-vivo kinetic constants from omics data (measured enzyme concentrations, metabolite concentrations, and fluxes); on the other hand, we may adjust and complete empirical data to construct models with consistent parameters and metabolic states. This may involve predictions of physiological metabolite concentrations [39,40] or predictions of metabolite and enzyme concentrations based on resource allocation principles [41,42,43].

Hence, in order to fit kinetic constants, we also need to reconstruct consistent metabolite concentrations and enzyme concentration from known metabolic fluxes and other available data. To reduce the uncertainties, we may further impose constraints by physical laws, including thermodynamic relations (Wegscheider conditions and Haldane relationships) between kinetic constants [1,6] and relations between kinetic constants and metabolic variables (e.g., the fact that flux directions follow from equilibrium constants and metabolite concentrations). In addition, we can use prior distributions (for kinetic constants, metabolite concentrations, or enzyme concentrations); and we can use measured (in vitro) parameter values as additional data. However, one problem remains. The resulting estimation problems are typically non-convex, allowing for multiple local optima, and global optima may be hard to find.

Here we propose a method for estimating consistent kinetic constants and metabolic states based on heterogeneous kinetic, metabolomics, and proteomics data, where metabolic fluxes must be known from measurements or previous calculations. This method, called Model Balancing, uses the following input data: kinetic constants, metabolite and enzyme concentrations in a number of metabolic states, and metabolic fluxes from the same metabolic states. Unlike Flux Balance Analysis (FBA), Model Balancing is not restricted to stationary fluxes, since Michaelis-Menten kinetics can also be used in dynamic simulations if the quasi-steady state approximation holds; however, the fluxes must be thermodynamically feasible. Except for the fluxes, no data are strictly required, but any additional data will make the estimates more precise. From these data, model balancing determines kinetic parameters and state variables (metabolite and enzyme concentrations) that respect the constraints, agree with prior distribution and data, and agree with the rate laws defined in the model. For the estimation, we maximise the Bayesian posterior density, which is the probability density function of all parameters given the priors and the measured data. Maximum-likelihood estimation can be obtained by the use of flat priors (i.e., wide and uniform distribution functions that have essentially no effect on the posterior). Among the terms in the logarithmic posterior, there is one that penalises enzyme concentrations that are lower than suggested by priors and data. If we neglect this term (or sufficiently scale it down), the posterior score becomes strictly convex, and the maximum-posterior problem has a single solution and can be solved by gradient descent. The convexity relies on two main assumptions: (i) all fluxes are predefined and thermodynamically consistent (i.e., infeasible cycle fluxes must be excluded) and (ii) kinetic constants and metabolite concentrations are described in log-scale. Model balancing builds upon other methods for model construction: Parameter Balancing [21,24] and Enzyme Cost Minimisation (ECM) [43], which are reviewed in the discussion section.

## 2. Materials and Methods

A reconstruction of kinetic constants and metabolic states, based on kinetic models and Bayesian estimation, was outlined in [20]. Here we implement this programme under the assumption that the fluxes are known. Building on [43], we show that this assumption makes the problem nearly convex.

### 2.1. Metabolic Model and Statistical Estimation Model

**Figure 1:**
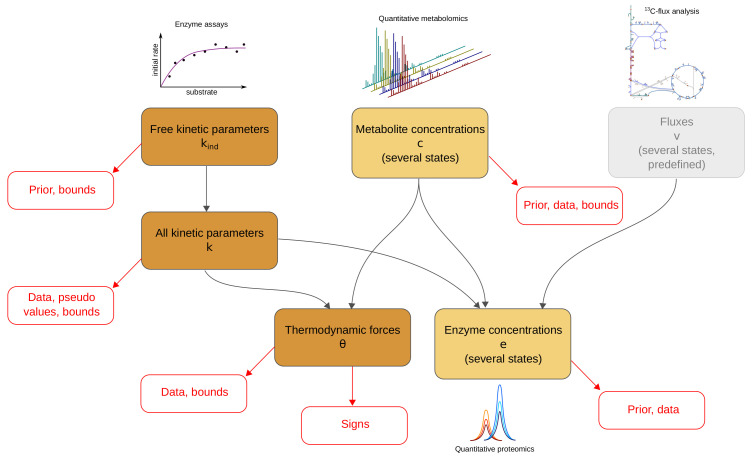
Model balancing relies on a dependency schema for kinetic constants and state variables. All kinetic constants are described in logarithmic scale. A subset of kinetic constants determines all other kinetic constants through linear relationships. If kinetic constants, metabolite concentrations, and fluxes are known, the enzyme concentrations can be computed from rate laws and fluxes: each enzyme concentration is a convex function of the (logarithmic) kinetic constants and metabolite concentrations. The signs of thermodynamic forces are constrained by the flux directions.

The model balancing problem is depicted in Figure 1 (for mathematical symbols see Supplementary Materials Table S1). We consider a kinetic metabolic model with logarithmic kinetic (and thermodynamic) constants (e.g., equilibrium constants, catalytic constants and Michaelis–Menten constants) in a vector q=lnp. We further assume a number of metabolic states *s*, characterised by flux vectors $v^{\left(s\right)}$, metabolite concentration vectors $c^{\left(s\right)}$, and enzyme concentration vectors $e^{\left(s\right)}$. These states may be stationary (with steady-state fluxes) or non-stationary (e.g., snapshots from a dynamic time course) and the fluxes must be thermodynamically feasible. In each state, the fluxes $v_{l}^{\left(s\right)}=e_{l}^{\left(s\right)}\;k_{l}\left(p,c^{\left(s\right)}\right)$ depend on enzyme concentrations $e_{l}^{\left(s\right)}$, metabolite concentrations $c_{i}^{\left(s\right)}$, and catalytic rates $k_{l}$. A typical rate law k(c), for a uni-uni reaction, is given by the reversible Michaelis–Menten kinetics, where $v=\frac{k_{\mathrm{cat}}^{+}\;s/K_{s}-k_{\mathrm{cat}}^{-}\;p/K_{p}}{1+s/K_{S}+p/K_{p}}$ [6]. In general, we use modular rate laws [6]. The vector of thermodynamic forces depends on metabolite concentrations as $\theta =\ln k_{\mathrm{eq}}-S^{⊤}\;\ln c$, where S is the stoichiometric matrix. In thermodynamically feasible rate laws, these forces determine the flux directions $\mathrm{sign}\left(v_{l}\right)=\mathrm{sign}\left(\theta_{l}\right)$ (where vanishing fluxes are always allowed). This sign constraint holds for any thermodynamically feasible rate laws. For convenience, reversible rate laws can also be written in the factorised form [44] $v=k_{\mathrm{cat}}^{+}\cdot \eta^{\mathrm{rev}}\left(c\right)\cdot \eta^{\mathrm{sat}}\left(c\right)$ with unitless efficiency terms $\eta^{\mathrm{rev}}=1-e^{-\theta (c)}$ (for thermodynamic reversibility) and $\eta^{\mathrm{sat}}$ (for enzyme saturation and allosteric regulation) between 0 and 1, depending on metabolite concentrations. With efficiency terms close to 1, k(c) approaches its maximal value $k_{\mathrm{cat}}$.

Due to physical laws [1,6], the kinetic constants and state variables in our model depend on each other: each rate law contains forward and backward catalytic constants and Michaelis–Menten constants, and all these parameters may depend on each other via Haldane relationships and Wegscheider conditions. Only activation and inhibition constants are independent of all other kinetic constants. To deal with these dependencies, we use a schema (see Figure 1) with a set of basic kinetic parameters (independent equilibrium constants, Michaelis–Menten constants, and velocity constants) that are physically independent and determine all remaining kinetic constants (Figure 1, top left, and Supplementary Materials). Notably, thermodynamic forces and enzyme concentrations appear as dependent variables. By inverting the rate laws, we obtain the enzyme demand function

(1) $$\begin{array}{c} e_{l}^{\left(s\right)}=\frac{v_{l}^{\left(s\right)}}{k_{l}\left(p,c^{\left(s\right)}\right)}, \end{array}$$

a function of kinetic constants, metabolite concentrations, and fluxes. We can also describe the enzyme demand as the flux, multiplied by a reaction time $\tau_{l}=1/k_{l}$. Importantly, the functions (1) as well as their logarithms are convex in the space spanned by q=lnp and x=lnc (see Supplementary Materials).

### 2.2. Model Balancing

In model balancing, $k_{\mathrm{cat}}$ values are not estimated separately, but together with other kinetic constants and with metabolite profiles, enzyme profiles, and thermodynamic forces in a number of metabolic states (for simplicity, we refer to all the kinetic/thermodynamic constants and the state variables together as “model variables”). In this way, full use can be made of prior knowledge and heterogeneous data. The data for all model variables may be available, but uncertain and incomplete. Only the fluxes are assumed to be known. Besides dependencies and data, we may use prior distributions and impose upper and lower bounds on model parameters and on metabolite and enzyme concentrations. All kinetic constants and concentrations – but not the thermodynamic forces and fluxes – are described by their natural logarithms. In model balancing, we distinguish between basic variables (basic kinetic constants and metabolite concentrations) and dependent variables (dependent kinetic constants, enzyme concentrations and thermodynamic forces). The basic variables are constrained by predefined ranges and, indirectly, by bounds on dependent variables. The resulting solution space is a convex polytope, on which a Bayesian posterior distribution is defined. Here, we compute the posterior mode (with maximum-likelihood estimation as a special case), but posterior sampling [45] would also be possible. For details, see Supplementary Materials.

Model balancing follows the ideas outlined in [20]. Starting from a Gaussian prior density p(x) for metabolite log-concentrations x, we take the negative logarithm, omit constant terms, and obtain the *prior loss score*

(2) $$\begin{array}{ccc} P(x) & = & \frac{1}{2}{\left(x-{\bar{x}}_{\mathrm{pr}}\right)}^{⊤}{\Sigma_{\mathrm{pr}}^{x}}^{-1}\left(x-{\bar{x}}_{\mathrm{pr}}\right)=\mathrm{quad}\left(x-{\bar{x}}_{\mathrm{pr}},\Sigma_{\mathrm{pr}}^{x}\right) \end{array}$$

for metabolite log-concentrations. The shorthand $\mathrm{quad}\left(a,\Sigma \right)=\frac{1}{2}a^{⊤}\Sigma^{-1}a$ is used for convenience, and the inverse covariance matrix ${\Sigma_{\mathrm{pr}}^{x}}^{-1}$ is called precision matrix. The vector x may contain metabolite log-concentrations from one or several metabolic states. If we treat log-scaled kinetic constants (q), metabolite concentrations (x), and enzyme concentrations (z=lne) as variables with independent priors, we obtain the preprior score

(3) $$\begin{array}{ccc} P^{☆}\left(q,x,z\right) & = & \mathrm{quad}\left(q-{\bar{q}}_{\mathrm{pr}},\Sigma_{\mathrm{pr}}^{q}\right)+\mathrm{quad}\left(x-{\bar{x}}_{\mathrm{pr}},\Sigma_{\mathrm{pr}}^{x}\right)+\mathrm{quad}\left(z-{\bar{z}}_{\mathrm{pr}},\Sigma_{\mathrm{pr}}^{z}\right). \end{array}$$

The name “preprior” and the star ${}^{☆}$ remind us that q, x, and z still appear as separate variables, despite their dependency in the model. Similarly, using data for q, x, and z, we define the “pre-likelihood score”

(4) $$\begin{array}{ccc} L^{☆}\left(q,x,z\right) & = & \mathrm{quad}\left(q-{\bar{q}}_{\mathrm{lk}},\Sigma_{\mathrm{lk}}^{q}\right)+\mathrm{quad}\left(x-{\bar{x}}_{\mathrm{lk}},\Sigma_{\mathrm{lk}}^{x}\right)+\mathrm{quad}\left(z-{\bar{z}}_{\mathrm{lk}},\Sigma_{\mathrm{lk}}^{z}\right). \end{array}$$

The vectors ${\bar{x}}_{\mathrm{lk}}$ and ${\bar{z}}_{\mathrm{lk}}$ and matrices $\Sigma_{\mathrm{lk}}^{x}$ and $\Sigma_{\mathrm{lk}}^{z}$ contain the mean values and covariances for measurement data. For simplicity, we assume that each state variable has been measured exactly once; a formula for the general case with missing and duplicate values is given in Supplementary Materials. By adding the functions (3) and (4), we obtain the *preposterior score* $R^{☆}\left(q,x,z\right)=P^{☆}\left(q,x,z\right)+L^{☆}\left(q,x,z\right)$, and by combining the quadratic functions (see [20,21]), we obtain the formula

(5) $$\begin{array}{ccc} R^{☆}\left(q,x,z\right) & = & \mathrm{quad}\left(q-{\bar{q}}_{\mathrm{po}},\Sigma_{\mathrm{po}}^{q}\right)+\mathrm{quad}\left(x-{\bar{x}}_{\mathrm{po}},\Sigma_{\mathrm{po}}^{x}\right)+\mathrm{quad}\left(z-{\bar{z}}_{\mathrm{po}},\Sigma_{\mathrm{po}}^{z}\right) \end{array}$$

with covariance matrix and mean vector

(6) $$\begin{array}{ccc} \Sigma_{\mathrm{po}}^{x} & = & {\left[{\Sigma_{\mathrm{pr}}^{x}}^{-1}+{\Sigma_{\mathrm{lk}}^{x}}^{-1}\right]}^{-1}\\ {\bar{x}}_{\mathrm{po}} & = & \Sigma_{\mathrm{po}}^{x}\;\left[{\Sigma_{\mathrm{pr}}^{x}}^{-1}\;{\bar{x}}_{\mathrm{pr}}+{\Sigma_{\mathrm{lk}}^{x}}^{-1}\;{\bar{x}}_{\mathrm{lk}}\right]. \end{array}$$

for metabolite concentrations in x, and analogous formulae for q and z. Equation (6) is based on the fact that when data and prior terms are combined, their precision matrices are additive and the posterior mean is a weighted sum of prior and likelihood means.

Until here, all score functions are convex functions: the prior score $P^{☆}$, likelihood score $L^{☆}$, and posterior score $R^{☆}$ are convex in q, x, and z, and since non-flat priors are used, prior and posterior scores are strictly convex. However, as we remember, the enzyme concentrations are not free variables, but dependent on metabolite concentrations and fluxes. Therefore, the vector z can be eliminated from the formulae, and the same holds for dependent kinetic constants in the vector q. From the enzyme demand function Equation (1), we obtain the function z(q,x). By inserting this function into Equation (4) and merging x and the independent parameters in q into a vector y, we obtain the three score functions as functions of y alone:

(7) $$\begin{array}{ccc} \mathrm{Prior}\;\mathrm{loss}\;P(y) & = & \mathrm{quad}\left(y-{\bar{y}}_{\mathrm{pr}},\Sigma_{\mathrm{pr}}^{x}\right)+\mathrm{quad}\left(z\left(y\right)-{\bar{z}}_{\mathrm{pr}},\Sigma_{\mathrm{pr}}^{z}\right)\\ \mathrm{Likelihood}\;\mathrm{loss}\;L(y) & = & \mathrm{quad}\left(y-{\bar{y}}_{\mathrm{lk}},\Sigma_{\mathrm{lk}}^{x}\right)+\mathrm{quad}\left(z\left(y\right)-{\bar{z}}_{\mathrm{lk}},\Sigma_{\mathrm{lk}}^{z}\right)\\ \mathrm{Posterior}\;\mathrm{loss}\;R(y) & = & \mathrm{quad}\left(y-{\bar{y}}_{\mathrm{po}},\Sigma_{\mathrm{po}}^{x}\right)+\mathrm{quad}\left(z\left(y\right)-{\bar{z}}_{\mathrm{po}},\Sigma_{\mathrm{po}}^{z}\right). \end{array}$$

In contrast to our pre-scores, these score functions depend only on the basic variables in y. To maximise the posterior density, we now need to

MinimiseR(y)in the polytope of feasible vectors y,

where the vector y describes the metabolite profiles and basic kinetic constants.

### 2.3. A Convex Version of the Score Functions

Will this problem still be convex? Convex optimality problems are numerically favourable, and methods like parameter balancing and ECM profit from this. Thus, are the prior, likelihood, and posterior losses (7) convex functions in y? In all three cases, the first term is a quadratic function of y and therefore convex. Thermodynamic forces are linear functions of y and therefore adding them to the score would still keep it quadratic. The second term is composed of a convex function quad(·) and a convex function z(y), but this does not make it convex. If it consisted of an inner convex function and an outer *non-decreasing* function, it would be convex, but in fact the outer function quad(·) contains terms that can be decreasing.

**Figure 2:**
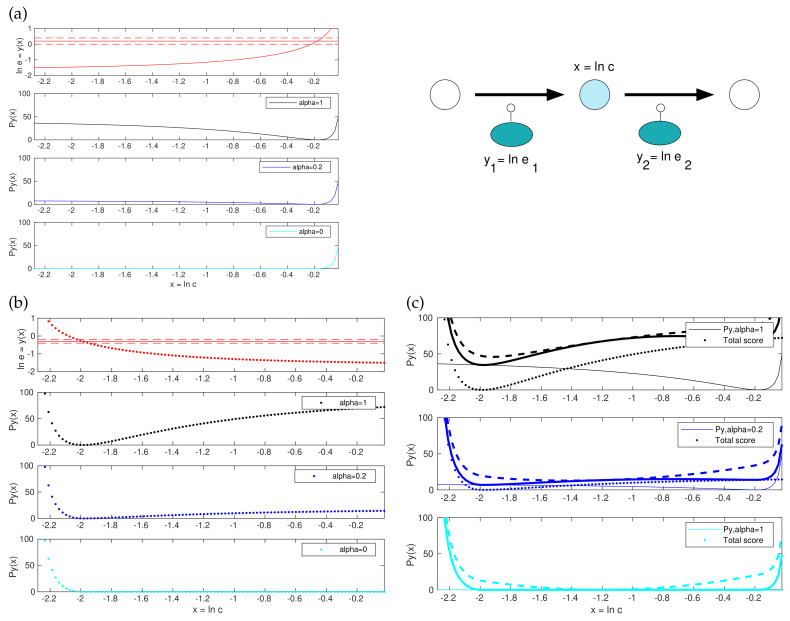
Model balancing and its convex variants. Top right: Example model. Given the fluxes and external metabolite concentrations, the log concentration *x* determines the enzyme log concentrations $y_{1}$ and $y_{2}$. (**a**) Posterior score for the enzyme level in reaction 1. Top: the log enzyme level $y_{1}$ depends on the metabolite log concentration *x* (solid curve). Horizontal lines show the preposterior mean (solid) and standard deviation (dashed). Black line: the posterior score term is zero where $y_{1}$ matches the preposterior mean and increases for smaller and larger values. Since the left part of the curve is negatively curved, the function is non-convex. In the plots below, this left part is decreased by a factor α=0.2 (dark blue) or removed completely by setting α=0 (cyan). The resulting last curve is convex; (**b**) same as (**a**), for reaction 2. The posterior score has its minimum at a lower *x* value than for reaction 1; (**c**) Posterior score for *x*. Top: The enzyme posterior scores for reaction 1 and 2 (solid and dotted black curves from (**a**,**b**)) are added (thick solid line). By further adding the metabolite term (which is strictly convex), we obtain the posterior score (dashed line), which is non-convex. In the case of α=0.2 (dark blue), the enzyme term is still non-convex, but the total posterior score becomes convex. In the case α=0, the enzyme term is convex, and the total posterior score is convex as well.

However, a small modification suffices to make the functions convex (see Figure 2 and Supplementary Materials). The posterior score term for enzyme levels, given by $\mathrm{quad}(z\left(y\right)-{\bar{z}}_{\mathrm{post}},\Sigma_{\mathrm{po}}^{z})$ with a diagonal matrix $\Sigma_{\mathrm{po}}^{z}$, is a sum of quadratic functions of the form $\frac{1}{2}{\left(z_{lt}\left(y\right)-\bar{z_{lt}}\right)}^{2}/\sigma_{lt}^{2}$. Each of these functions consists of a decreasing branch (for $z_{lt}\left(y\right)-\bar{z_{lt}}<0$) and an increasing branch (for $z_{lt}\left(y\right)-\bar{z_{lt}}\geq 0$) If we replace the decreasing branches with zeros, we obtain a non-decreasing, truncated function ${\mathrm{quad}}_{0}\left(z\left(y\right)-{\bar{z}}_{\mathrm{post}},\Sigma_{\mathrm{po}}^{z}\right)$. Using this function instead of quad makes the model balancing problem convex (see Figure 2 and Supplementary Materials). Instead of omitting the decreasing branch, we scale it down by a factor α∈[0,1], leading to a relaxed function ${\mathrm{quad}}_{\alpha }\left(z\left(y\right)-{\bar{z}}_{\mathrm{post}},\Sigma_{\mathrm{po}}^{z}\right)$. The stringency parameter α has a simple interpretation: whenever a predicted enzyme concentration is below the preposterior mean, the corresponding score term is omitted (for α=0), or scaled down (for 0<α<1). Effectively, replacing α=1 by a smaller value increases the assumed preposterior standard deviation of (logarithmic) enzyme concentrations, selectively for values below the preposterior mean (setting α<1 increases the assumed standard deviations by a factor $1/\sqrt{\alpha }$, e.g. by a factor of ≈3 when α is set to 0.1). For sufficiently small values of α, and for α=0 specifically, the entire model balancing problem becomes strictly convex. However, the penalty for low enzyme levels is under-represented. To summarise, we can choose between three versions of the model balancing problem: (i) the original version (α=1) that penalises small or large enzyme levels symmetrically and is non-convex; (ii) a truncated version (α=0) that is strictly convex and can be implemented by Disciplined Convex Programming [46]; here the estimates of enzyme levels may be too low, entailing poor estimates in kinetic constants; (iii) and a relaxed version (0<α<1) that is convex for small values of α; in our tests, we usually obtained good results at α≈0.1 and above.

### 2.4. Details and Variants of Model Balancing

How can we deal with inactive reactions? If a flux vanishes, then either the catalyzing enzyme’s level has to be zero, or the reaction is at thermodynamic equilibrium. In our method, enzyme concentrations are treated on log-scale and cannot be zero. One could redefine the problem using absolute enzyme concentrations (rather than log-scaled) but that would be harder to solve numerically. Furthermore, exact zero fluxes (e.g. from FBA results) may not be reliable and enforcing them would likely over-constrain the other model variables. Instead, we omit that specific reaction from the scoring function (only for metabolic states where the flux through it is zero) and ignore the measured enzyme level even if it is provided in the prior.

Kinetic constants estimated from omics data may be poorly determined or even non-identifiable (see Supplementary Materials). Due to the usage of priors, non-identifiability does not mean that variables are ill-determined: the method will return a posterior with a unique mode. However, the estimates of “non-identifiable” variables are not supported by data, but completely depend on prior means. Since the priors have a strong impact on model balancing results, the usage of informative priors [21,47] is key to our method. To obtain realistic priors for all our model variables, we started from priors in [12,24,43] and modified them based on experimental data (see Supplementary Materials).

We explored variants of model balancing with additional terms in the score functions, including pseudo values for dependent variables and regularisation terms for $c/K_{M}$ ratios (see Supplementary Materials). In our tests, these variants did not improve the results. Another variant concerns the usage of cross-covariances. The preposterior for kinetic constants in a model (equilibrium constants, forward and backward $k_{\mathrm{cat}}$ values, and $K_{M}$ values) is a multivariate normal distribution, characterised by a covariance matrix. For simplicity, this matrix can be replaced by a block matrix in which cross-covariances between different types of variables are omitted. This does not affect the Haldane relationships, because all physical dependencies between variables are maintained through the parameterisation by independent basic variables. In our tests, omitting the cross-covariances had little effects on the estimation results. Other variants of the algorithm—with priors and data for thermodynamic forces, and terms reducing fast metabolite changes in time series—are described in the Supplementary Materials.

## 3. Results

### 3.1. Model Balancing

Model Balancing is a Bayesian method for fitting metabolic models to kinetic and omics data, where the fluxes must be given and consistent metabolite and enzyme concentrations are determined from incomplete, uncertain data for metabolite and enzyme concentrations. Mathematically, the problem resembles Enzyme Cost Minimisation (ECM) [43], which assumes a kinetic model with known parameters and predefined fluxes and optimises metabolite and enzyme concentrations for a minimal biological cost. In model balancing, concentrations are not optimised but fitted to data, along with the kinetic constants, by maximising the posterior density. Metabolite and enzyme concentrations are described by natural logarithms. Furthermore, metabolite concentrations are restricted to a thermodynamically feasible solution space, which ensures that Equation (1) yields positive results for the enzyme concentrations. To illustrate the basic idea, let us first assume that all kinetic constants are known, and consider a single metabolic state. For the metabolite log-concentration vector x=lnc, we assume Gaussian priors (with mean vector ${\bar{x}}_{\mathrm{pr}}$ and a diagonal covariance matrix $\Sigma_{\mathrm{pr}}^{x}$) and lower and upper bounds (possibly different for each metabolite). Likewise, for the enzyme log-concentration vector z=lne, we assume Gaussian priors with mean vector ${\bar{z}}_{\mathrm{pr}}$ and covariance matrix $\Sigma_{\mathrm{pr}}^{z}$). The possible metabolite log-profiles x form a convex polytope $P_{x}$ in log metabolite space [43]. The polytope is defined by physiological upper and lower bounds and by thermodynamic constraints, depending on flux directions and equilibrium constants. The metabolite log-concentrations $x_{i}$, our free variables, determine the enzyme concentrations $e_{l}$ through Equation (1), and the resulting enzyme concentrations and log-concentrations are convex functions on the metabolite polytope (Supplementary Materials). To define an estimation problem, we construct the polytope as our solution space and use prior, likelihood and posterior functions on this polytope to estimate metabolite concentrations and corresponding enzyme concentrations.

Model balancing has the same favourable properties as this simplified problem. A description of the algorithm, including the convexity proof for the function lne(q,x), is given in Supplementary Materials. Following Liebermeister and Klipp [1], the kinetic constants can be parameterised by independent basic constants. Instead of a metabolite vector x (as in our simplified problem), we consider a larger vector y, containing the metabolite log-concentrations for all metabolic states and the vector of log kinetic constants. Along with metabolite and enzyme data, data for kinetic constants may be used (for instance, equilibrium constants $K_{\mathrm{eq}}$ from thermodynamic calculations), and consistent values need to be estimated. Since kinetic constants cannot change between metabolic states and since kinetic constants and state variables are estimated together, all these variables become effectively coupled and need to be estimated in one go.

All terms in the posterior score, except for the terms for enzyme levels, are convex. Non-convexity and convexity are illustrated in Figure 2, for a simple model with two enzymes and one adjustable metabolite log-concentration *x*; for simplicity, the other metabolite concentration as well as kinetic constants are assumed to be known. Subfigures (a) and (b) show how the enzyme demand for the two reactions (as a function of *x*) gives rise to non-convex score functions (while their truncated α=0 version is convex). By adding these two curves and a convex score term for *x*, we obtain the posterior score shown in subfigure (c). In the original problem (top), it is non-convex; with a lower α (centre), the enzyme terms are non-convex, but the total score is convex due to the convex term for *x*; and in the truncated α=0 version, all terms are convex and so the total score is known to be convex as well.

Implementations for Matlab and Python are freely available (see Supplementary Materials and [48]). As a test case for model balancing, we consider a model of *E. coli* central metabolism and corresponding metabolite, enzyme, and kinetic data from [43] (for details, see [48]). We studied estimation scenarios with artificial and experimental data (data from [43] from one metabolic state, aerobic growth on glucose). Smaller example models, including model balancing results, are provided along with our code. In all cases, the model balancing algorithm was run with the same settings, priors, and bounds and without pseudo values and regularisation for $c/K_{M}$. The results below stem from a non-convex version of model balancing with strictness parameter α=0.5.

### 3.2. Tests with Artificial Data

For our tests with artificial data, we first generated “true” kinetic constants and state data (metabolite concentrations, enzyme concentrations, and fluxes). To obtain realistic distributions of kinetic constants, we used the same broad distributions that were also used as a prior. By doing this, we assume that our priors are realistic. Otherwise, the reconstruction may be slightly worse than suggested by our tests with artificial data. State variables were generated from the kinetic model (with the “true” kinetic constants) by choosing enzyme concentrations and external metabolite concentrations at random (for small test models) or close to realistic metabolic states (for the *E. coli* model) and computing the steady states (details in Supplementary Materials). Now the aim was to reconstruct the true values for six simulated states from (noise-free or noisy) artificial data. We considered different scenarios (Figure 2 in Supplementary Materials) in which variables were either fitted (metabolite and enzyme concentrations, and kinetic constants given as data) or predicted from the other data (“unknown” kinetic constants).

**Figure 3:**
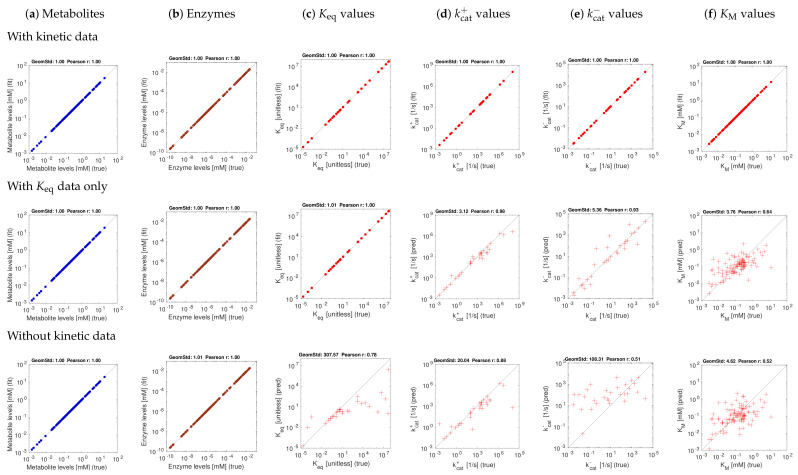
Model balancing results for *E. coli* model with artificial data (stringency parameter α=0.5). The model structure is shown in [48]. Each subfigure shows “true” artificial values (*x*-axis) versus reconstructed values (*y*-axis). The quality of fits or predictions is quantified by geometric standard deviations and by Pearson correlations on logarithmic scale. (**a**) Metabolite concentrations; (**b**) enzyme concentrations; (**c**–**f**) equilibrium constants, $k_{\mathrm{cat}}$ values, and Michaelis–Menten constants. The three rows show different estimation scenarios (see Supplementary Materials Figure S2). Upper row: simple scenario S1 (noise-free artificial data, data for all kinetic constants). Centre row: scenario S1K (noise-free artificial data, kinetic data given only for equilibrium constants). Lower row: scenario S2 (noise-free artificial data, no data for kinetic constants). Depending on the scenario, kinetic constants are either fitted (dots) or predicted (crosses).

**Figure 4:**
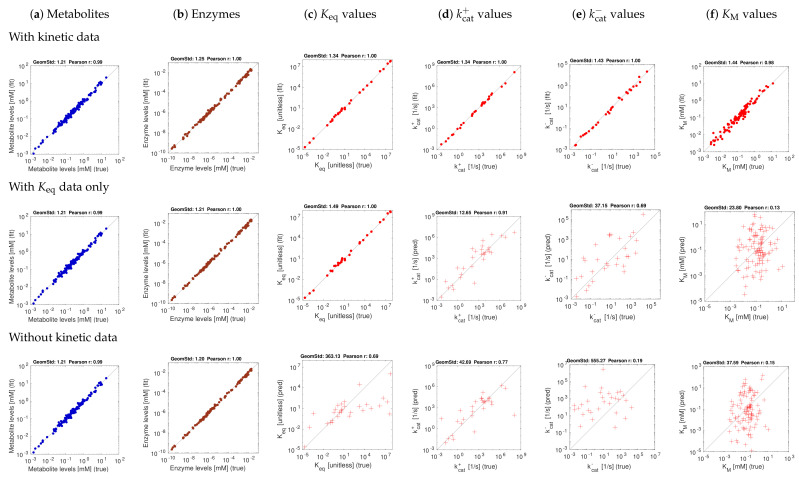
Model balancing results for *E. coli* model with artificial data (stringency parameter α=0.5). Same as Figure 3, but with noisy kinetic data and noisy state data.

Figure 3 and Figure 4 show the model balancing results with artificial data and different combinations of noise-free or noisy kinetic and noise-free or noisy state data. We chose a stringency value of α=0.5 (results for other stringency values can be found on github.com/liebermeister/model-balancing; accessed on 20 October 2021). Results for noise-free kinetic and state data are shown in Figure 3. Scatter plots compare true and fitted variables (metabolite concentrations, enzyme concentrations, and different types of kinetic constants). Deviations from the diagonal line indicate estimation errors. Subfigures show different types of variables (columns) and estimation scenarios (rows). In the top row, data for all kinetic constants were provided; in the centre row, only data for equilibrium constants were provided, and in the bottom row, no kinetic data were used at all. Depending on whether data for equilibrium constants and other kinetic constants were used, scatter plots represent either fitting scenarios in which kinetic constants only adjusted (dots), or prediction scenarios (crosses) in which kinetic constants were determined from state data. The quality of fits or predictions is quantified by Pearson correlations (in log-scale) and geometric standard deviations (i.e. the exponent of the root mean square of the residuals in log-scale).

The first scenario (top row) assumes ideal conditions, with noise-free and complete kinetic and state data. The reconstructions are perfect, with small deviations arising from conflicts between priors and data. The other rows show estimation results using equilibrium constants only (centre row) or using no kinetic data at all (bottom row). Metabolite and enzyme data were used in all cases, either without or with noise. The predictions of $k_{\mathrm{cat}}$ and $K_{M}$ values from complete and noise-free data are rather good. To assess the effect of noise, we next used state data (metabolite concentrations, enzyme concentrations, and fluxes) with a noise level of about 20 percent (geometric standard deviation 1.2) and noisy kinetic data, with a geometric standard deviation of 1.5. With noisy data, the estimates become worse (Figure 4f), and especially $K_{M}$ values are predicted poorly. Using equilibrium constants as data improves the results, and $k_{\mathrm{cat}}$ values can be reconstructed more reliably (Figure 4d,e).

To explore how our stringency parameter α affects the results, we ran model balancing (for the *E. coli* example and several smaller pathway models) at different values of α. The results were as expected: at α=0, enzyme concentrations tend to be underestimated, while this effect typically disappeared at α values above 0.1. Generally, our tests with artificial data show that model balancing can adjust noisy data sets: relatively small changes in the data suffice to obtain a consistent set of kinetic and state data. Second, they show that information about $k_{\mathrm{cat}}$ values can be extracted from state data. It turns out that $K_{M}$ values are much harder to reconstruct. Given that real metabolic data are noisy, estimating in-vivo values from omics data remains difficult, and adjusting in-vitro kinetic constants to realistic in-vivo values remains the main application of model balancing.

### 3.3. Model Fitting with Experimentally Measured Data

After this test, we balanced the *E. coli* model with experimentally measured data from the literature. We used the in-vitro kinetic constants collected in [43] (“original kinetic data”) and, for comparison, a parameter-balanced version of the same data set (“balanced kinetic data”).

**Figure 5:**
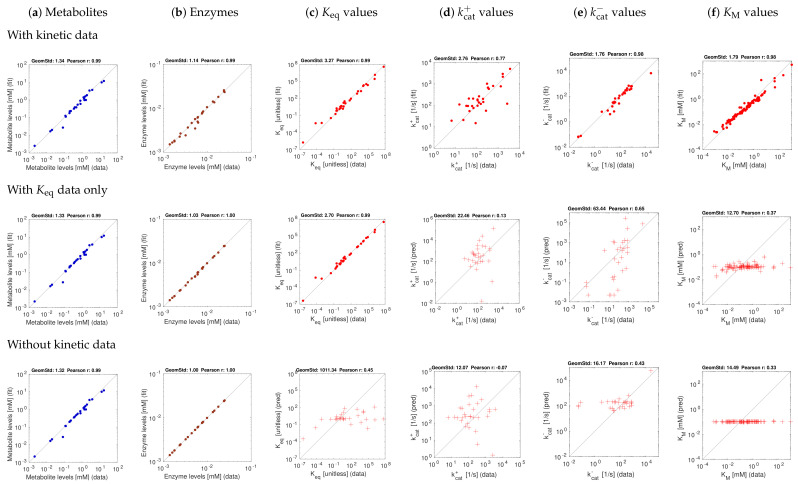
Results for *E. coli* central metabolism with experimental data (aerobic growth on glucose). The kinetic data stem from previous parameter balancing based on in-vitro data. **Top**: estimation using kinetic data. **Centre**: model fit using equilibrium constants as data. **Bottom**: estimation without usage of kinetic data. Metabolite, enzyme, and kinetic data were taken from [43]. A stringency parameter α=0.5 was used. The fact that our kinetic data were obtained from parameter balancing, based on the same network model and the same priors, leads to a bias. However, a test with original in-vitro kinetic data (and thus fewer data points, not shown) yielded similar results. Predicted kinetic constants may be validated by cross-validation; the results can be expected to lie in between the results from our “fitting” (dots) and “prediction” scenarios (crosses).

Figure 5 shows results for one metabolic state, aerobic growth on glucose, with a stringency value of α=0.5. Since the true kinetic constants and state variables are unknown, the reconstructed kinetic constants, metabolite concentrations and enzyme concentrations are plotted against the available data, where dots refer to fits and crosses refer to predictions (where kinetic data were used only for validation). In a first test, we used a complete set of kinetic constants obtained by parameter balancing (Figure 5). With these constants used as data (top row, red dots), slight adjustments were sufficient to obtain a consistent kinetic model. Using only equilibrium constants as data (centre row) yields reasonable predictions of the $k_{\mathrm{cat}}$ values. In the bottom row, both equilibrium constants and other kinetic constants were predicted, but the correspondence to in-vitro values is relatively small.

## 4. Discussion

### 4.1. Model Balancing in Relation to Other Methods

To integrate kinetic and omics data into consistent metabolic models, uncertainties and missing data need to be handled. Here we showed that the Bayesian framework proposed in [20] leads to nearly convex estimation problems if the metabolic fluxes are known. Depending on data availability and quality, model balancing can either be used to infer kinetic constants from omics data or to make data complete, consistent, and more precise.

Kinetic constants have been inferred by various methods. Kinetic profiling is easy to implement, and its computational effort is low, but it provides only lower bounds. Model fitting, in contrast, can integrate different types of data (including $K_{\mathrm{eq}}$ values and in-vitro $k_{\mathrm{cat}}$ values) and can estimate $k_{\mathrm{cat}}$ values even if enzymes do not reach their maximal rates in experiments. In contrast to methods like SIMMER [16], which considers single reactions (and requires complete data for each of them), model balancing can transfer information between different parts of a network. Maud [49] is a similar approach for integrating flux, metabolite, enzyme and kinetic data. Maud uses Markov Chain Monte Carlo (MCMC) sampling combined with an ODE solver for determining steady states and with posterior sampling. One of its advantages over model balancing is that metabolic flux data are not required for running the algorithm. However, since running an ODE for each sample point requires more computational resources, it is expected to be less scalable.

**Figure 6:**
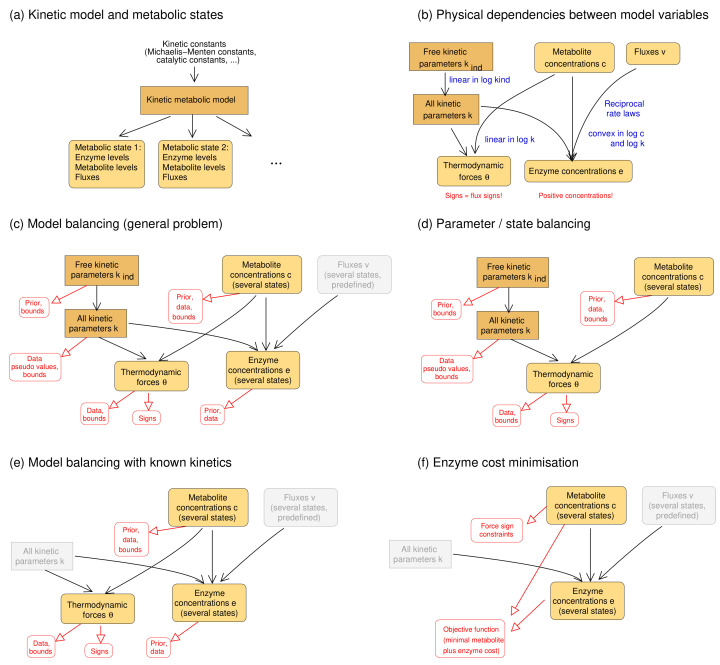
Estimation methods for kinetic constants and metabolic states. (**a**) Kinetic model and metabolic states. A model is parameterised by kinetic constants (e.g., equilibrium constants, catalytic constants, and Michaelis–Menten constants) and is used to generate a number of metabolic states (characterised by enzyme concentrations, metabolite concentrations, and fluxes). States may be stationary (with steady-state fluxes) or not (e.g., states during dynamic time courses); (**b**) dependencies between kinetic constants and state variables (see Figure 1); (**c**) model balancing. Kinetic constants and metabolite concentrations (for several states) are the free variables of a statistical model. Dependent kinetic constants, thermodynamic driving forces, and enzyme concentrations (bottom) are the dependent variables, and fluxes (top right) are predefined. Priors and data may be used in the estimation. The other graphics show (**d**) parameter balancing [21,24] for kinetic data and metabolite concentrations; (**e**) model balancing with given kinetic constants; (**f**) enzyme cost minimisation [43], in which enzyme and metabolite concentrations are optimised for a low enzyme and metabolite cost.

Parameter fitting are often non-convex, especially if thermodynamically feasible flux distributions are to be estimated [50] (noisy flux data will compromise the estimation results, but as long as the fluxes can be thermodynamically realised, model balancing remains applicable). To obtain problems that are nearly convex, model balancing borrows ideas from existing methods, Parameter Balancing (PB) and Enzyme Cost Minimisation (ECM) (see Figure 6): it assumes that fluxes are predefined and uses logarithmic kinetic constants and metabolite concentration as the free variables. We note that estimating the fluxes themselves would require an additional level of optimisation which, typically, is hard to solve numerically [50].

- *Parameter balancing.* Parameter balancing determines consistent kinetic constants from kinetic and thermodynamic data. Unlike model balancing, it does not use rate laws or flux data. All multiplicative constants (such as Michaelis–Menten constants or catalytic constants) are described by log-values, which leads to a linear regression problem. The equilibrium constants are parameterised directly by standard chemical potentials rather than independent variables that can be adjusted if needed [24]. With Gaussian priors and measurement errors (in log-scale), likelihood and posterior terms are quadratic and convex. Parameter balancing can handle either kinetic and thermodynamic constants (“kinetic parameter balancing”), metabolite concentrations and thermodynamic forces (“state balancing”), or kinetic constants and metabolic states (“state/parameter balancing”). With known signs of thermodynamic forces, defined by the flux directions, parameter balancing can predict thermodynamically feasible kinetic constants and metabolite concentrations. While its optimisation takes place on the same set as in model balancing, it does not consider rate laws and cannot be used to fit kinetic constants to flux data. As a post-processing step, balanced kinetic constants can be adjusted to rate laws and flux data, but this works only for a single metabolic state so, unlike in model balancing, data from multiple states cannot be combined.
- *Enzyme cost minimisation.* Enzyme cost minimisation (ECM) [43] predicts optimal enzyme and metabolite concentrations in kinetic models with given parameter values. ECM determines metabolite and enzyme concentrations that realise predefined fluxes at a minimal cost, for instance, at a minimal total enzyme and metabolite concentration. The optimisation is carried out in (log-)metabolite space. In contrast to parameter balancing, ECM assumes given kinetic constants and optimises a biological cost rather than a goodness of fit. With given rate laws, the cost function (a weighted sum of enzyme and metabolite concentrations) is convex in log-metabolite space.

Model balancing combines aspects of these two methods. Just like parameter balancing, it estimates model parameters and steady-state variables; kinetic constants and metabolite concentrations are treated as basic variables that define a parameter/concentration polytope as the solution space. From ECM, we inherit the assumption that fluxes are given and that enzyme concentrations are convex functions of the metabolite log-concentrations. Model balancing relies on two additional insights: the fact that (both absolute and logarithmic) enzyme concentrations are convex functions on the combined solution space of kinetic and metabolic variables, and the fact that most of the terms in the posterior score are convex, while non-convex terms (limiting enzyme concentrations from below) can be omitted or weakened by a tunable prefactor.

In summary, all three methods—parameter balancing, ECM, and model balancing—use a convex (or nearly convex) score function in a convex solution space, a high-dimensional polytope in the space of log kinetic constants, metabolite log-concentrations, or both (depending on the method). To construct it, we define a box by upper and lower bounds and add linear constraints describing dependencies. The solution polytope for model balancing is obtained from the polytopes of the other methods by taking their Cartesian product and removing infeasible regions, in which constraints between kinetic constants and metabolite concentrations are violated (shown in Supplementary Materials Figure S1). Since all variables are estimated together, information about one variable (bounds, priors, or data) can improve the estimates of other variables as well. In model balancing, this holds not only for kinetic constants, but also includes metabolic states.

### 4.2. Model Balancing in Practice

Model balancing can integrate different types of variables (defined by dependency schemas), integrate different types of knowledge (network structure, data, priors, and constraints). Our tests show that small adjustments of kinetic and omics data suffice to obtain consistent models and metabolic states. While precise data for state variables and equilibrium constants would allow for a partial reconstruction of $k_{\mathrm{cat}}$ and $K_{M}$ values, noisy omics data lead to poor prediction results. While this may be disappointing, model balancing can still tell us what information about kinetics can be obtained from data with realistic noise levels, and whether other data (or improvements in measurement accuracy) can improve the results. This may have a bearing on experimental design.

Depending on the types of data available (kinetic constants, metabolite concentrations, or enzyme concentrations), model balancing can be applied in different ways.

- *Inferring missing data types* If fluxes and two of the data types are given, the third type can be estimated. For example, we may estimate in-vivo kinetic constants from metabolite concentrations and enzyme concentrations; we may estimate metabolite concentrations from enzyme concentrations and enzyme kinetics; or we may estimate enzyme concentrations from metabolite concentrations and enzyme kinetics. If the data were complete and precise, the third type of variables could be directly computed, and model balancing would not be necessary. But when data are uncertain and incomplete, model balancing allows us to infer the missing data while completing and adjusting the others.
- *Adjusting omics data to obtain complete, consistent metabolic states* Given a model with known kinetic constants, we can translate metabolite and enzyme data into complete, consistent metabolic states. Again, fluxes must be given and thermodynamically realisable with the assumed equilibrium constants and metabolite bounds. We can even estimate metabolic states without any enzyme or metabolite data: in this case, model balancing predicts plausible states with the given fluxes, relying on priors for enzyme or metabolite concentrations.
- *Imposing thermodynamic constraints and bounds on data* To build consistent metabolic models, we may collect data for kinetic and state variables and apply model balancing. The resulting kinetic constants and state variables satisfy the rate laws, agree with physical and physiological constraints, and resemble data and prior values. Above we used this to construct a physically and biologically plausible model of *E. coli* central metabolism. Posterior sampling (as in [16]) might be used to assess uncertainties in model parameters.

In all cases, diagnostic plots on networks can help curate data, e.g. by displaying how data points were adjusted. In summary, estimating in-vivo $k_{\mathrm{cat}}$ values from metabolomics and proteomics data remains difficult: in our tests with artificial data, data with realistic noise levels led to unreliable predictions. However, adjusting existing in-vitro kinetic constants to in-vivo values seems to be feasible: the results in Figure 5, top, show that mild adjustments of in-vitro kinetic constants suffice to obtain consistent models that explain omics data. This type of model adjustment, to improve metabolic models, could be a main application.

Model balancing can be applied to heterogeneous data. Even if few or no data are available (except for the fluxes), it can be used to build model ensembles. In our tests with noise-free artificial data, model balancing performed well in prediction, and with noisy data it can still be used to make models consistent. Unlike its predecessor methods, model balancing is not a convex problem unless the terms that penalise low enzyme concentrations are omitted or made very small. We expect that non-convexity arises, in particular, if enzyme data values are overly high, carrying large errors, but if small error bars are wrongly assumed. To find the right value of α, we recommend starting from small values and gradually increasing them until the enzyme fitting error becomes acceptable. In our tests, this was typically the case above α=0.1. Another limitation of model balancing is its calculation speed, which ranges between minutes and several hours for the *E. coli* example shown. The number of variables increases with model size and the number of metabolic states, which impacts memory requirements and calculation time. Because it is simple, kinetic profiling can be much faster. However, model balancing is probably faster than standard model fitting approaches, such as Maud [49] that compute steady states by numerical integration.

In this paper the kinetic constants and state variables in a metabolic model were scored by a posterior distribution, and we computed the posterior mode. Posterior sampling, as a next step, would allow us to estimate the posterior mean, variance, covariances and marginal distributions of kinetic constants and state variables. Moreover, entire parameter sets could be sampled and model ensembles could be constructed. To simplify sampling, the posterior can be approximated by a multivariate Gaussian distribution, obtained from the posterior mode and the Hessian matrix of the posterior score in this point. Such a sampling procedure has been implemented for parameter balancing, but not yet for model balancing. We hope that our formulation of the problem, with a nearly convex posterior score, will facilitate posterior sampling and will pave the way to efficient sampling algorithms, which may also include a sampling of fluxes [50].

## Data Availability

All data and code are freely available at github.com/liebermeister/model-balancing (accessed on 20 October 2021) (matlab code) and gitlab.com/elad.noor/model-balancing (python version) (accessed on 20 October 2021). Documentation can be found at model-balancing.readthedocs.io (accessed on 20 October 2021).

## Supplementary Materials

The following are available online at https://www.mdpi.com/article/10.3390/metabo1010000/s1, Figure S1: Solution space in model balancing, Figure S2: Model balancing: estimation scenarios with artificial data, Table S1: Mathematical symbols used in model balancing.

## Abbreviations

The following abbreviations are used in this manuscript:

ECM	Enzyme Cost Minimisation
FBA	Flux Balance Analysis
