# Supplementary material for: Model Balancing: A Search for In-Vivo Kinetic Constants and Consistent Metabolic States
Source: Metabolites. 2021 Oct 29;11(11):749. doi: 10.3390/metabo11110749 (PMC8621975; doi:10.3390/metabo11110749)
Supplement: Supplementary file 1 [file metabolites-11-00749-s001.zip › metabolites-1407957- supplementary.pdf]

Model balancing: a search for in-vivo kinetic constants and consistent metabolic states

Supplementary Materials

Wolfram Liebermeister[1,*] and Elad Noor[2]

[1]Université Paris-Saclay, INRAE, MaIAGE, 78350, Jouy-en-Josas, France
[2]Department of Plant and Environmental Sciences, Weizmann Institute of Science, Israel
[*]Corresponding author. Email: wolfram.liebermeister@inrae.fr

1 The model balancing problem

To define a model balancing problem, we consider all model variables (kinetic constants and state variables), describe their dependencies by a dependency schema, and define score functions based on priors, likelihood, and posterior distributions (see Table 1).

1.1 Model variables, constraints, and cost scores

Model variables The model variables are split into independent "basic" variables and dependent "derived" variables based on the following rules. (i) With kinetic constants described in log-scale, all dependencies are linear. Among the kinetic constants, some are treated as basic variables, while all others are treated as derived. There is some freedom in choosing the basic variables, and our matlab and python implementations of model balancing use different choices. Here we describe the choice that is implemented in matlab[1], with independent equilibrium constants, Michaelis-Menten constants, and velocity constants (geometric means of forward and backward catalytic constants) as basic variables, and dependent equilibrium constants and catalytic constants as derived variables. (ii) For each metabolic state s, we consider a metabolite log-concentration vector $\mathbf{x}^{(s)} = \ln \mathbf{c}^{(s)}$, an enzyme concentration vector $\mathbf{z}^{(s)} = \ln \mathbf{e}^{(s)}$, and a flux vector $\mathbf{v}^{(s)}$. Vectors from different metabolic states are not arranged into data matrices, but are merged into large vectors $\mathbf{x}, \mathbf{z}$, and $\mathbf{v}$. The fluxes are predefined. (iii) Since enzyme concentrations depend on kinetic constants, metabolite concentrations, and fluxes, they are treated as dependent variables. (iv) Thermodynamic driving forces depend on equilibrium constants and metabolite concentrations, and are therefore dependent. Thus, basic kinetic constants and metabolite concentrations are the only basic variables. (v) The flux directions determine the signs of thermodynamic driving forces, leading to linear constraints between (logarithmic) equilibrium constants and metabolite concentrations. Altogether, we obtain the following variables and dependencies.

[1] In parameter balancing, Gibbs free energies of formation are the independent parameters behind the equilibrium constants. The Gibbs free energies of formation determine all equilibrium constants, but in a redundant way. While they could be used in model balancing too, an alternative (minimal) set of variables is given by the equilibrium constants of a subset of the reactions l, where the columns of the stoichiometric matrix (for all external and internal metabolites) corresponding to these reactions form a maximal set of linearly independent columns.

**State variables**		
Metabolic flux	$v_l^{(s)}$	Reaction l, in state s
Metabolic log-concentration	$x_i^{(s)} = \ln c_i^{(s)}$	Metabolite i, in state s
Enzyme log-concentration	$z_l^{(s)} = \ln e_l^{(s)}$	Reaction l, in state s
**Kinetic constants**		
Log kinetic constants	$\mathbf{q}, \mathbf{q}^{\rm ind}, \mathbf{q}^{\rm dep}$	
Equilibrium constants	$\mathbf{k}_{\rm eq}, \mathbf{k}_{\rm eq}^{\rm ind}, \mathbf{k}_{\rm eq}^{\rm dep}$	
Catalytic constants	$k_{{\rm cat},l}^{+}, k_{\rm cat,l}^{-}$	Reaction l
Velocity constants	$K_{\rm V,l} = \sqrt{k_{{\rm cat},l}^{+}, k_{\rm cat,l}^{-}}$	Reaction l
Michaelis-Menten constants	$K_{M,li}$	Reaction l, reactant i
Activation constants	$K_{A,li}$	Reaction l, activator i
Inhibition constants	$K_{I,li}$	Reaction l, inhibitor i
**Estimation score functions**		
Prior prescore	$P^{\star}(\mathbf{x}, \mathbf{z}, \mathbf{q})$	
Likelihood prescore	$L^{\star}(\mathbf{x}, \mathbf{z}, \mathbf{q})$	
Posterior prescore	$R^{\star}(\mathbf{x}, \mathbf{z}, \mathbf{q})$	
Prior score	$P(\mathbf{x}, \mathbf{q}^{\rm ind})$	
Likelihood score	$L(\mathbf{x}, \mathbf{q}^{\rm ind})$	
Posterior score	$R(\mathbf{x}, \mathbf{q}^{\rm ind})$	

Table 1: Mathematical symbols used in model balancing

1. **Basic variables** By default, our basic variables comprise (i) the independent kinetic constants in log-scale (independent equilibrium constants $\ln K_{\rm eq}^{\rm ind}$, Michaelis-Menten constants $\ln K_{\rm M}$, allosteric activation constants $\ln K_{\rm A}$, allosteric inhibition constants $\ln K_{\rm I}$, and velocity constants $\ln K_{\rm V}$) in a vector

$$\mathbf{q}^{\rm ind} = \begin{pmatrix} \ln \mathbf{k}_{\rm eq}^{\rm ind} \\ \ln \mathbf{k}_{\rm V} \\ \ln \mathbf{k}_{\rm M} \\ \ln \mathbf{k}_{\rm A} \\ \ln \mathbf{k}_{\rm I} \end{pmatrix}, \quad (1)$$

and (ii) the metabolite log-concentrations from one or several metabolic states s, forming metabolite vectors $\mathbf{x}^{(s)} = \ln \mathbf{c}^{(s)}$. We obtain a vector of free variables

$$\mathbf{y} = \begin{pmatrix} \mathbf{x}^{(1)} \\ \mathbf{x}^{(2)} \\ .. \\ \mathbf{q}^{\rm ind} \end{pmatrix}. \quad (2)$$

With n_p independent kinetic constants, n_m metabolites, and n_s metabolic states, the vector contains $n_p + n_m\, n_s$ free variables.

2. **Derived variables** We consider three types of dependent variables: dependent kinetic constants, enzyme concentrations, and thermodynamic forces. (i) The dependent kinetic constants on log-scale (dependent equilibrium constants $\ln K_{\rm eq}^{\rm dep}$, forward catalytic constants $\ln k_{\rm cat}^{+}$), and backward catalytic constants $\ln k_{\rm cat}^{-}$) form a vector

$$\mathbf{q}^{\rm dep} = \begin{pmatrix} \ln \mathbf{k}_{\rm eq}^{\rm dep} \\ \ln \mathbf{k}_{\rm cat}^{+} \\ \ln \mathbf{k}_{\rm cat}^{-} \end{pmatrix}. \quad (3)$$

This vector can be computed from $\mathbf{q}^{\rm ind}$ with the help of a linear function $\mathbf{q}^{\rm dep} = \mathbf{M}^{\rm dep}\,\mathbf{q}^{\rm ind}$. The dependency matrix $\mathbf{M}^{\rm dep}$ follows from the stroichiometric matrix as described in [1]. Similarly, the vector $\mathbf{q}$ of all kinetic constants is given by a linear formula

$$\mathbf{q} = \begin{pmatrix} \mathbf{q}^{\rm ind} \\ \mathbf{q}^{\rm dep} \end{pmatrix} = \begin{pmatrix} \mathbf{I} \\ \mathbf{M}^{\rm dep}_{\rm ind} \end{pmatrix} \mathbf{q}^{\rm ind} = \mathbf{M}^{\rm all}_{\rm ind}\,\mathbf{q}^{\rm ind}. \tag{4}$$

If we focus on only those constants that actually appear in the rate laws (catalytic constants and Michaelis constants), we can list them in a smaller vector $\mathbf{q}^{\rm mode}$, which is then related to the basic kinetic constants via a similar formula $\mathbf{q}^{\rm mod} = \mathbf{M}^{\rm mod}_{\rm ind}\,\mathbf{q}^{\rm ind}$. (ii) The thermodynamic forces are computed by the linear formula

$$\boldsymbol{\theta}^{(s)} = \ln \mathbf{k}_{\rm eq} - \mathbf{S}^{\top}\,\mathbf{x}^{(s)} \tag{5}$$

or briefly $\boldsymbol{\theta} = \mathbf{M}^{\theta}\,\mathbf{y}$ with the stoichiometric matrix $\mathbf{S}$ (for all metabolites) and a matrix $\mathbf{M}^{\theta}$ obtained from the network structure. (iii) Based on rate laws and using Eq. (1), the enzyme concentration vectors $\mathbf{e}^{(s)}$ are given by

$$e^{(s)}_l = \frac{v^{(s)}_l}{k_l(\mathbf{q}, \mathbf{x}^{(s)})}. \tag{6}$$

For a wide range of rate laws, both $e^{(s)}_l$ and its logarithm $z^{(s)}_l = \ln e^{(s)}_l$ are convex functions in $(\mathbf{q}, \mathbf{x})$. Moreover, Eq. (6) yields positive enzyme concentrations on the entire solution space $\mathbf{x}$ (see below), except when fluxes vanish: if $v^{(s)}_l = 0$, we directly set $e^{(s)}_l = 0$ (and omit $\ln e^{(s)}_l$ from the list of model variables).

Constraints The solution space for our free variables is defined by two sorts of constraints. First, feasible ranges on all variables except for the enzyme concentrations[2]

$$\mathbf{q}^{\rm min} \le \mathbf{q} \le \mathbf{q}^{\rm max}, \qquad \mathbf{x}^{\rm min} \le \mathbf{x}^{(s)} \le \mathbf{x}^{\rm max}, \qquad \boldsymbol{\theta}^{\rm min} \le \boldsymbol{\theta}^{(s)} \le \boldsymbol{\theta}^{\rm max}, \tag{7}$$

where s is the index for metabolic states. Second, the driving forces must be positive along the flux directions, and so the known fluxes define the signs of all driving forces. For reactions with non-zero fluxes $v^{(s)}_l \neq 0$, we obtain the thermodynamic constraints

$$v^{(s)}_l\,\theta^{(s)}_l > 0, \tag{8}$$

which (together with Eq. (5)) define linear constraints for the vector $\mathbf{y}$. In reactions with zero fluxes, the driving forces remain unconstrained (unless we know, for some other reasons, that reactions are in chemical equilibrium). Together our constraints can be written as

$$\mathbf{A}\,\mathbf{y} \le \mathbf{b}, \tag{9}$$

with a matrix $\mathbf{A}$ and a vector $\mathbf{b}$ obtained from reaction stoichiometries and flux directions. These constraints Eq. (7) and (9) define a convex solution polytope $\mathcal{P}$. Each polytope point $\mathbf{y}$ describes a feasible set of model parameters and metabolic states (i.e. states with positive driving forces). Conversely, any feasible set of kinetic constants and metabolic states (respecting all bounds and constraints) corresponds to a point in the polytope.

[2]Positive enzyme concentrations are ensured by the other formulae. With thermodynamically feasible rate laws, the enzyme concentrations $e^{(s)}_l(\mathbf{q}, \mathbf{x}^{(s)})$ for active reactions, Eq. (6), are positive and convex on the entire solution polytope $\mathcal{P}$. Upper bounds on individual enzyme concentrations (or on weighted sums of enzyme concentrations) would yield non-linear inequality constraints, giving the solution polytope a curved shape. However, since these constraints are convex, the resulting solution set would still be convex. Such constraints are not considered here, but they could be used without any problems. Lower bounds on enzyme concentrations (or on absolute metabolite concentrations), in contrast, would make the solution space (on log-scale), and therefore the optimality problem non-convex.

Geometry of the solution space Model balancing determines the vectors $\mathbf{q}$ and $\mathbf{x}$ at the same time. The resulting vector $\mathbf{y}$ lives in a high-dimensional polytope (see Figure 1): the polytope resembles a Cartesian product of the polytopes for these single vectors, but due to thermodynamic constraints between kinetic constants and metabolite concentrations, some parts of this Cartesian product must be removed. To see how the metabolite spaces for several metabolic states are combined, let us return to our simplified model balancing problem from section 2.2. We can solve this problem separately for each state, and this is also the easiest way to solve the problem. But we can also fit all metabolic states simultaneously by one big regression model. Each metabolite profile $\mathbf{x}^{(s)}$ must lie in a metabolite polytope $\mathcal{P}_{\mathbf{x}}^{(s)}$. If the flux directions in all metabolic states are the same, these polytopes are identical; if fluxes change their directions, the metabolite polytopes $\mathcal{P}_{\mathbf{x}}^{(s)}$ will differ. If we merge all vectors $\mathbf{x}^{(s)}$ into a vector $\mathbf{x}$, the solution polytope for this vector will be higher-dimensional and will be given by the Cartesian product $\prod_s \mathcal{P}_{\mathbf{x}}^{(s)}$. As before, we can consider the prior, likelihood, and posterior (for all metabolic states) as functions on this solution space. Since the metabolic states are independent, the prior, likelihood, and posterior functions can be split into products of priors, likelihoods, and posteriors for the single states, confirming that the estimation problems could have been solved separately.

Prior and likelihood terms Finally, we define prior, likelihood and posterior scores. The posterior is obtained from prior and likelihood terms. Kinetic constants, metabolite concentrations, and enzyme concentrations are considered in log-scale and described by Gaussian priors and likelihood terms on this scale. For the basic kinetic constants, we use a correlated prior described by independent prior terms (for independent kinetic constants) and from pseudo values (for dependent kinetic constants). While pseudo values are introduced to define correlated priors, they can be treated in practice as additional data points (see [1]). For the state variables (metabolite log-concentrations $x_i^{(s)}$ and enzyme concentrations $z_i^{(s)}$) , we assume uncorrelated Gaussian priors (i.e. log-normal priors for the concentrations themselves). Similarly, the logarithmic data values $x_{i,\mathrm{data}}^{(s)}$ and $z_{l,\mathrm{data}}^{(s)}$ in the likelihood term, are assumed to be independent and follow Gaussian distributions around the true values.

1.2 Convex score functions

Convexity proof The truncated score function $R_0(\mathbf{y})$ (that is, the posterior loss, using a truncated function quad_0 for the enzyme term) is convex. To see this, we note:

1. The logarithmic flux-specific enzyme demand (or "reaction time")

$$\ln \tau_l(\mathbf{x}^{(s)}, \mathbf{q}) = \ln \frac{1}{k(\mathbf{x}^{(s)}, \mathbf{q})} \tag{10}$$

   (for reaction l and metabolic state s) is convex in $(\mathbf{x}^{(s)}, \mathbf{q})$ in the entire feasible space for $(\mathbf{x}^{(s)}, \mathbf{q})$ and for all factorisable rate laws [2]. For $\mathbf{x}^{(s)}$ alone, this has been shown in [3]. For the combined vector $(\mathbf{x}^{(s)}, \mathbf{q})$, convexity holds because Eq. (10), with factorisable rate laws, can be entirely decomposed into functions allowed in Disciplined Convex Programming [4]. For details, see section 4 and [5].

2. The enzyme log concentration $\ln e_l(\mathbf{x}^{(s)}, \mathbf{q}) = \ln[\tau_l(\mathbf{x}^{(s)}, \mathbf{q})\, v_l^{(s)}]$ is linear in $\tau_l(\mathbf{x}^{(s)}, \mathbf{q})$ and therefore a convex function in $(\mathbf{x}^{(s)}, \mathbf{q})$.

3. In the prior score, likelihood score, and posterior score

$$\begin{aligned} P(\mathbf{x}) &= \mathrm{quad}(\mathbf{q} - \bar{\mathbf{q}}_{\mathrm{pr}}, \boldsymbol{\Sigma}^{\mathbf{q}}_{\mathrm{pr}}) + \mathrm{quad}(\mathbf{x} - \bar{\mathbf{x}}_{\mathrm{pr}}, \boldsymbol{\Sigma}^{x}_{\mathrm{pr}}) + \mathrm{quad}(\mathbf{z}(\mathbf{q}, \mathbf{x}) - \bar{\mathbf{z}}_{\mathrm{pr}}, \boldsymbol{\Sigma}^{z}_{\mathrm{pr}}) \\ L(\mathbf{x}) &= \mathrm{quad}(\mathbf{q} - \bar{\mathbf{q}}, \boldsymbol{\Sigma}^{\mathbf{q}}_{\mathrm{lk}}) + \mathrm{quad}(\mathbf{x} - \bar{\mathbf{x}}_{\mathrm{lk}}, \boldsymbol{\Sigma}^{x}_{\mathrm{lk}}) + \mathrm{quad}(\mathbf{z}(\mathbf{q}, \mathbf{x}) - \bar{\mathbf{z}}, \boldsymbol{\Sigma}^{z}_{\mathrm{lk}}) \\ R(\mathbf{x}) &= \mathrm{quad}(\mathbf{q} - \bar{\mathbf{q}}_{\mathrm{po}}, \boldsymbol{\Sigma}^{\mathbf{q}}_{\mathrm{po}}) + \mathrm{quad}(\mathbf{x} - \bar{\mathbf{x}}_{\mathrm{po}}, \boldsymbol{\Sigma}^{x}_{\mathrm{po}}) + \mathrm{quad}(\mathbf{z}(\mathbf{q}, \mathbf{x}) - \bar{\mathbf{z}}_{\mathrm{po}}, \boldsymbol{\Sigma}^{z}_{\mathrm{po}}). \end{aligned} \tag{11}$$

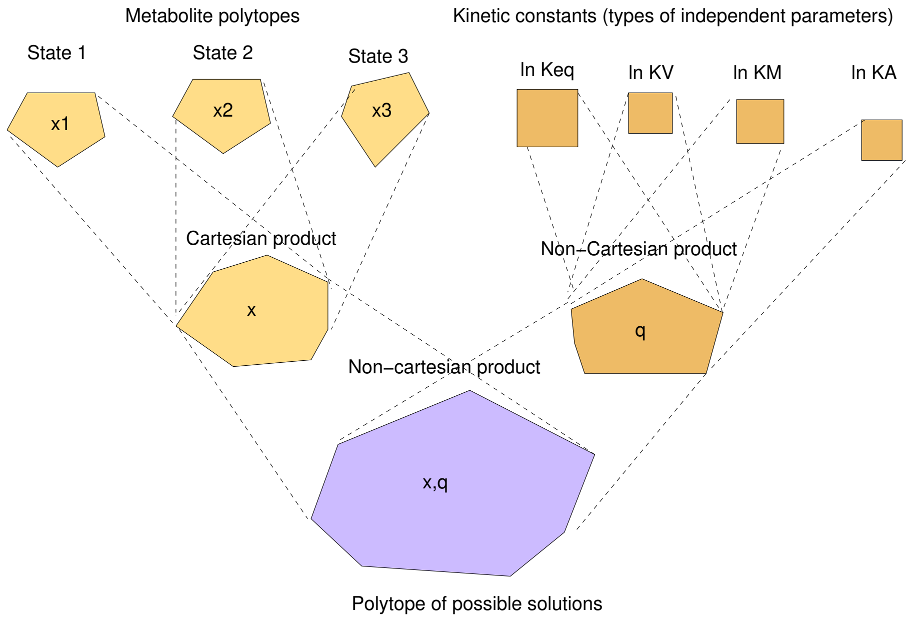

Figure 1: Solution space in model balancing. The basic model variables (kinetic constants and metabolite concentrations on log-scale) are constrained by physiological bounds and thermodynamic constraints dependent on flux directions. Together, these inequality constraints define a solution space, a high-dimensional polytope in the space of log-variables (bottom). Geometrically, this polytope is a "non-Cartesian product" between a metabolite polytope and a kinetic constant polytope (centre): a Cartesian product from which some parts are removed by extra constraints. The metabolite polytope is a Cartesian product of the metabolite polytopes for single metabolic states (which may differ due to different flux directions); the polytope of kinetic constant is a (non-Cartesian) product of boxes for different types of constants (top right).

the first terms are quadratic in $\mathbf{q}$ and independent of $\mathbf{x}$, while the second terms are quadratic in $\mathbf{x}$ and independent of $\mathbf{q}$. Both terms are therefore convex in $(\mathbf{x}^{(s)}, \mathbf{q})$. The third terms are quadratic in $\mathbf{z}(\mathbf{q}, \mathbf{x})$ and are not convex. However, if we replace the function quad by its truncated version quad_0, each component can be written as a sum of non-decreasing functions of $z_l^{(s)}(\mathbf{q}, \mathbf{x})$, which are convex in $(\mathbf{q}, \mathbf{x})$, so the terms are convex in $(\mathbf{q}, \mathbf{x})$ themselves.

Relaxed and convex score functions The original model balancing problem, minimising $R(\mathbf{y})$, is in general a non-convex problem. To make it convex, we can modify the posterior score for enzyme concentrations. We assume an uncorrelated prior for enzyme concentrations, i.e. a separate Gaussian prior for each reaction l and each metabolic state s. If we do this, the second term becomes a sum of the form

$$\text{quad}(\mathbf{z}(\mathbf{x}) - \bar{\mathbf{z}}, \mathbf{\Sigma}^z) = \sum_{ls} G(z_l(\mathbf{x}^{(s)}) - \bar{z}_{ls}, \sigma_{ls}), \tag{12}$$

with a one-dimensional quadratic function $G(x - \bar{x}, \sigma) = \frac{1}{2}\frac{(x-\bar{x})^2}{\sigma^2}$. Next, we split this function into $G = G_+ + G_-$, where $G_+ = G$ only if $x \geq a$ (and 0 otherwise) and $G_- = G$ only if $x < a$ (and 0 otherwise), and define the relaxed quadratic function

$$\text{quad}_\alpha(\mathbf{z}(\mathbf{x}) - \bar{\mathbf{z}}, \mathbf{\Sigma}^z) = \sum_{ls} G_+(z_l(\mathbf{x}^{(s)}) - \bar{z}_{ls}, \sigma_{ls}) + \alpha \sum_{ls} G_-(z_l(\mathbf{x}^{(s)}) - \bar{z}_{ls}, \sigma_{ls}). \tag{13}$$

Note that by setting $\alpha = 1$, we reobtain Eq. (12), by setting $\alpha = 0$, we obtain only the first term (with G_+), and with α values in between we can interpolate between these two cases. Importantly, since G_+ is an increasing function, the function $\text{quad}_\alpha(\mathbf{z}(\mathbf{x}) - \bar{\mathbf{z}}, \mathbf{\Sigma}^z)$ is a sum of non-decreasing functions $G_+(\cdot)$ of a convex function $\mathbf{z}(\mathbf{x})$

and therefore convex[3] in $\mathbf{x}$! We now define the relaxed prior loss $P_\alpha(\mathbf{x})$, the relaxed likelihood loss $L_\alpha(\mathbf{x})$, and the relaxed posterior loss $R_\alpha(\mathbf{x})$ by replacing quad by quad$_\alpha$ in the enzyme term. By varying α between 0 and 1, we can interpolate between the convex truncated problem and the non-convex, original problem. By choosing α small enough, the non-convex enzyme terms can be made arbitrarily small, and since the other terms are strictly convex, there will be some positive value $\alpha > 0$ below which the problem is always convex.

1.3 Additional regularisation terms

The posterior score function is based on data and prior beliefs about model variables. From a point of view of maximum-likelihood estimation, prior scores can be seen as regularisation terms, expressing preferences for certain values of model variables. Aside from the terms described in the paper, additional terms can be used to implement additional biological hypotheses.

- **Pseudo values.** If we assume independent priors for all basic variables in the dependency schema, this leads to a prior distribution of the dependent variables. However, this distribution may not match our expectations about the dependent variables. To correct this, we would have to introduce a prior for the dependent variables. While this is formally impossible (since their distribution is already defined via the basic variables), we may employ so-called pseudo values: hypothetical data values with broad error bars representing plausible ranges. Effectively, priors and pseudo values together define a dependent prior on the basic variables. In our test examples, pseudo values did not considerably improve the results, and in our code, they are not used by default.

- **Regularisation term for c/K_M ratios.** If a K_M value is much higher or lower than the corresponding typical metabolite concentration, this can have disadvantages for the cell: in the first case, enzyme is used inefficiently, while in the second case, the reaction is "inelastic" and a fluctuating substrate influx leads to large concentration fluctuations. If we assume that cells prevent these two cases, we may penalise, as a prior assumption, c/K_M ratios that differ too much from 1. To keep metabolite concentrations reasonably close to the respective K_M values, we added a quadratic term that scores the sum of log ratios $\ln c_{is}/K_{\mathrm{M}il}$ over each metabolite i, corresponding enzymes l, and metabolic states s. The term is supposed to prevent enzymes from operating in full saturation or fully in the linear range, and it can reduce – indirectly – concentration changes for each metabolite. The penalty term can be formulated as

$$T_{c/K_\mathrm{M}} = \sum_{ils} \frac{\beta^2}{2} \ln(c_{is}/K_{\mathrm{M}il})^2 \tag{14}$$

  with indices i (metabolite), l (reaction), s (sample), and a stringency parameter[4] β. Since this term is convex in our basic variables (and strictly convex in many of them) it improves the convexity of the overall problem. When we tried using this term with artificial data, the prediction of K_M values became worse; while this was partially expected (because our artificial data were not made to yield c/K_M ratios close to 1), we concluded that the term might do more harm than good, and did not use it in the remaining tests.

- **Priors and data for thermodynamic forces.** Thermodynamic forces, as model variables, may be expected to lie in some typical ranges, and sometimes measurement data may be available [6]. Priors or data for driving forces (not to be confused with priors for equilibrium constants) can be used to define new, force-related terms in each of the pre-scores ($P^\star$, $L^\star$, and $R^\star$), and as a consequence in P, L, and R. Since $\boldsymbol{\theta}$ is

[3] Remember that the enzyme log demand $\ln \mathbf{e}(\mathbf{x})$ is a convex function on the metabolite polytope [3] for a wide range of plausible rate laws.

[4] If we see this term as a prior, the stringency parameter β can be interpreted as $1/\mathrm{std}(\ln c/kM)$. Importantly, this is not equal to $1/\mathrm{std}_{geom}(c/K_\mathrm{M})$, but instead, to $1/\ln(\mathrm{std}_{geom}(c/K_\mathrm{M}))$. In theory, specific priors (and even data values) for each single c/kM ratio could also be used.

a linear function of $\mathbf{x}$, it adds another quadratic (and therefore convex) function of $\mathbf{x}$ to the existing term for $\mathbf{x}$. We did not use this feature so far, but it would be easy to implement.

- **Temporal correlations in metabolite concentrations** In measured time series, a metabolite concentration will not fluctuate randomly, but rather smoothly, and will therefore be correlated between subsequent timepoints. If we expect such correlations, we can implement this belief by a prior term for temporal changes, i.e. a penalty on temporal differences $\Delta_{t_i} \ln c = \ln c(t_(i+1)) - \ln c(t_i)$. To derive such a term, we may assume that each log metabolite concentration follows a diffusion process (Wiener process) in continuous time τ. The difference $\Delta_t \ln c(\tau) = \ln c(\tau) - \ln c(0)$ starts at $\Delta_t \ln c(\tau = 0) = 0$ and its standard deviation evolves like std$(\Delta_t \ln c(\tau)) = \sqrt{t/T_{\mathrm{char}}}$, with a characteristic time[5] T_{char}. Based on this model, we can define a quadratic penalty term of the form $T = \frac{\ln c(t+1) - \ln c(t)}{2\,\Delta\tau/T_{\mathrm{char}}}$. This penalty term is convex in $\ln c$ and can be included into model balancing.

- **Temporal correlations in enzyme levels** Since enzyme dynamics is slower than metabolite dynamics, it would be even more important to avoid variations of enzyme levels. In theory, we could do this by a similar term, penalising differences $z_l(t+1) - z_l(t)$ for each enzyme level $z_l = \ln e_l$ and each (sample) timepoint t. Unfortunately, such terms would be non-convex in our basic variables, for a simple reason: even if the z terms themselves were convex, then some of them would appear with a minus sign, which makes the term concave. To make all terms convex, z would have to be linear in $x = \ln c$, which is obviously not the case. Thus, to ensure smooth changes of enzyme levels, it is recommended not to add a penalty term, but to smoothen the enzyme data in advance (or, alternatively, to smoothen the enzyme preposterior means). To obtain the parameters for this smoothing a diffusion process (or a Kalman filter) can be applied, similar to the smoothing for metabolites.

2 Model balancing as a practical tool

2.1 Implementation

Matlab implementation A Matlab implementation including example models, data, and documentation is available at github.com/liebermeister/model-balancing. The file format for models and data (kinetic constants, fluxes, metabolite concentrations, protein concentrations) is SBtab [7] and metabolic networks can be defined in SBML [8] or SBtab files. Data structures describing model balancing problems can be exported as .mat files (for model balancing in matlab) or json (for model balancing in python).

Python implementation An indendent implementation in python features several numerical solvers, including a disciplined convex programming solver in cvxpy (for the case $\alpha = 0$). Code and documentation can be found at gitlab.com/elad.noor/model-balancing. Model balancing problems must be formulated as json files (including model structure and preposterior modes and variances). Such json files can be generated in matlab, and can be used to run direct comparisons between matlab and python solvers. For further details, see model-balancing.readthedocs.io

Comparison between matlab and python implementation We compared the results from our matlab and python implementations, which solve the same problem (defined in the same data structure) with different solvers. In a small test example, the results were largely identical, with minor discrepancies in some of the kinetic constants. In the *E. coli* model, there were larger discrepancies. A detailed comparison for two test models is shown in the github repository, folder `examples/graphics/matlab_python_comparison`.

[5] T_{char} is the time at which std$(\Delta_t \ln c(\tau)) = 1$, i.e. the geometric standard deviation of $c(\tau)$ is given by e.

2.2 Variants and simplifications

Model balancing can be adapted in various ways.

- **Model balancing without any data** If a type of data is not used, likelihood terms for this data type are omitted. Even without any data, priors will keep the results in biochemically plausible ranges.
- **Precisely known parameters** If certain parameters (e.g. the equilibrium constants) are precisely known, their values can be predefined (e.g. by treating them as data with very small standard errors).
- **Cross-covariances** As noted in the main article, the preposterior cross-covariances between different types of kinetic constants can be ignored, thus omitting some terms in the z-scores. In this case, the z-score for kinetic constants considers covariances within each type of variables, but not between types of variables. Our tests show that this has almost no effect on the results. We therefore omit these terms by default in the matlab implementation, and always in the python implementation (where it would have increased the size of the json files and increased the effort in disciplined convex programming). It is important to note that the Haldane relationships in our model depend on the parameterisation, not on the usage of cross-covariances; therefore, these relations still hold.
- **Cost terms for allosteric regulation** An allosteric activation with efficiency factor $\eta^{\rm reg} = \frac{c/K_A}{1+c/K_A}$ in the rate laws yields a cost factor $\frac{1+c/K_A}{c/K_A} = 1 + K_A/c$ in the formulae for enzyme demands. At constant metabolite levels, this corresponds to an extra enzyme demand $\Delta e_l = \frac{e_l\, K_{A,li}}{c_i}$ (where l and i denote the regulated reaction and the regulating metabolite, respectively). Similarly, an allosteric inhibition with efficiency factor $\eta^{\rm reg} = \frac{1}{1+c/K_I}$ yields a cost factor $1 + c/K_I$. If the metabolite levels are kept constant, this corresponds to an extra enzyme demand $\Delta e_l = \frac{e_l\, c_i}{K_{I,li}}$.
- **Irreversible rate laws** Model balancing also applies to models with irreversible rate laws. In an irreversible rate law, there are fewer kinetic constants (since backward catalytic constants, equilibrium constants, and velocity constants do not play a role); the forward kinetic constant is a free parameter, and no Haldane relationship is considered. Describing (some or all) rate laws as irreversible changes the structure of the kinetic dependency matrix $\mathbf{M}$.
- **Model parameterisations** Instead of independent equilibrium constants, standard chemical potentials may be used as independent parameters [1].
- **Initialising model balancing by parameter balancing or ECM** A preposterior for kinetic parameters may be obtained by previous parameter balancing, and pseudo values for metabolite and enzyme concentrations may be obtained by a previous ECM.
- **Penalising high concentrations** To penalise unrealistically high metabolite or enzyme concentrations, a regularisation term may be added, for example, proportional to the cost function considered in ECM.
- **Relative changes between metabolis states** Instead of containing absolute metabolite and enzyme concentrations, as assumed above, omics data may contain relative changes between metabolic states. To account for such data, a variant of the dependency schema might be considered: for each metabolite, we split the log-concentrations $\ln c_i^{(s)}$ into a reference value $\ln c_i$ and a deviation $\Delta \ln c_i^{(s)}$. Uncorrelated priors for these variables yield a meaningful correlated prior for the metabolite concentrations, and a similar splitting can be used for enzyme concentrations.
- **Data preprocessing by parameter balancing** In theory model balancing can be run in two steps, in which the first step is a simple parameter balancing problem: we consider only kinetic constants and fit them to kinetic data. The result is a multivariate Gaussian posterior for all log kinetic constants [1] that summarises

all data and prior knowledge about the kinetic constants. In the second step, we use this posterior as a prior for the kinetic constants and fit kinetic constants and model states (metabolite and enzyme concentrations) to metabolite and enzyme data. Since the kinetic data are already used to define the prior, they can be ignored in this part of the estimation. The calculation is equivalent to the method described in this paper. By processing the kinetic data separately, we can see what information is contained in these data, before combining them with metabolic data. Moreover, a kinetic "prior" that already captures the kinetic data may help us constrain the solution space for kinetic constants. This feature has not been implemented so far.

2.3 Measurement units

- **Measurement units** In metabolic modeling, different conventions exist about measurement units, and in principle, model balancing works for any of these conventions. However, in our implementation, priors and constraints are defined in a central file, which assumes the units mM (for metabolite and enzyme concentrations), mM/s for fluxes, and 1/s for $k_{\rm cat}$ values. When changing the units, this file must be modified.
- **Standard concentrations** In reaction thermodynamics, quantities such as equilibrium constants or Gibbs free energies of reaction are defined with respect to a standard concentration, which plays a role in reactions with unequal numbers of substrate and product molecules. Typically, flux balance models and kinetic ODE models use different conventions (1 M in the case of flux balance models, and 1 mM in the case of kinetic models). Our implementation allows for either of the conventions. To avoid errors, the convention must be stated in all input files containing thermodynamic data.
- **Compartments** Model balancing can be applied to compartment models; like in all models without compartments, fluxes are expressed as concentrations per time and the compartment sizes do not play a role.
- **Transport reactions** In transport reactions, the flux is defined as a concentration (of reaction events) per time. The definition of concentrations (e.g. in a membrane) reflects the concentration of enzyme molecules (in the same membrane).

2.4 Calculation details

Model balancing can be improved by a number of tricks and simplifications.

1. **Calculation of the posterior** To compute the posterior score from prior and likelihood scores (Eq. 6 for metabolite concentrations, and similar formulae for enzyme concentrations and kinetic constants), we need to invert a covariance matrix. This can be numerically expensive. To compute the posterior of the independent kinetic constants, we need to solve

$$\begin{aligned}\mathbf{\Sigma}^{\mathbf{q}}_{\rm ind,post} &= [\mathbf{\Sigma}^{\mathbf{q}}_{\rm ind,prior}{}^{-1} + \mathbf{M}^{\top}\,\mathbf{\Sigma}^{\mathbf{q}}_{\rm lk}{}^{-1}\mathbf{M}]^{-1} \\ \bar{\mathbf{q}}_{\rm ind,post} &= \mathbf{\Sigma}^{\mathbf{q}}_{\rm ind,post}\,[\mathbf{\Sigma}^{\mathbf{q}}_{\rm ind,prior}{}^{-1}\,\bar{\mathbf{q}}_{\rm ind,prior} + \mathbf{M}^{\top}\,\mathbf{\Sigma}^{\mathbf{q}}_{\rm lk}{}^{-1}\,\bar{\mathbf{q}}_{\rm lk}]\,. \end{aligned} \tag{15}$$

The calculation of $\mathbf{\Sigma}^{\mathbf{q}}_{\rm ind,prior}{}^{-1}$ and $\mathbf{\Sigma}^{\mathbf{q}}_{\rm lk}{}^{-1}$ (precision matrices for metabolite and enzyme concentrations) is easy because the original covariance matrices are diagonal and the matrices $\mathbf{D}$ select single vector elements. However, inverting the term in brackets may be hard. To speed up the calculation, we set $\mathbf{A} = \mathbf{\Sigma}^{\mathbf{q}}_{\rm ind,post}{}^{-1}$ and obtain the similar formulation

$$\begin{aligned}\mathbf{A} &= \mathbf{\Sigma}^{\mathbf{q}}_{\rm ind,prior}{}^{-1} + \mathbf{M}^{\top}\,\mathbf{\Sigma}^{\mathbf{q}}_{\rm lk}{}^{-1}\mathbf{M} \\ \bar{\mathbf{q}}_{\rm ind,post} &= \mathbf{A}^{-1}\,[\mathbf{\Sigma}^{\mathbf{q}}_{\rm ind,prior}{}^{-1}\,\bar{\mathbf{q}}_{\rm ind,prior} + \mathbf{M}^{\top}\,\mathbf{\Sigma}^{\mathbf{q}}_{\rm lk}{}^{-1}\,\bar{\mathbf{q}}_{\rm lk}]\,. \end{aligned} \tag{16}$$

Now the costly matrix inversion in the first equation is avoided, and the right-hand side in the second equation can be computed without explicitly computing the matrix inverse (e.g. by using the matrix left division operator \ in matlab). This calculation is faster and works for sparse matrices.

2. **Reactions with vanishing flux** If a reaction is always inactive – that is, if the flux is zero in all metabolic states – the kinetic constants for this reaction are ill-determined and the reaction should be removed from the model. But what if a reaction flux vanishes in some of the metabolic states? A vanishing flux can be caused by a vanishing enzyme level or by a vanishing thermodynamic force. However, for the purpose of model balancing, we assume that a vanishing flux always goes with a vanishing enzyme level. Therefore, whenever there is a zero flux, we set the enzyme level to zero in this metabolic state and ignore the corresponding term in the posterior score for enzyme levels.

3. **Divergence of enzyme concentrations close to polytope boundaries.** Enzyme concentrations (and therefore the likelihood function) increase very steeply close to thermodynamics-related polytope boundaries; to avoid numerical problems, a region close to the boundary may be excluded by extra constraints, or the log(log posterior) may be minimised instead of the log posterior. Here are some details. Each thermodynamic constraint defines a boundary of the solution polytope. Close to this boundary, an enzyme concentration goes to infinity and the likelihood function explodes. This steep increase can cause numerical problems during optimisation. To handle them, we may apply the logarithm function once more to the (likelihood or posterior) score, and use the resulting function as our minimisation objective. This new objective function will still go to infinity at polytope boundaries, but less steeply. The new objective function may be non-convex, but since it depends monotonically on a convex function, it will still have a single local minimum. A second way to avoid this problem is to exclude problematic regions close to the boundary by introducing some extra constraints. In practice. we can make all thermodynamic constraints a bit tighter, by requiring small, non-zero thermodynamic forces in every reaction [3].

2.5 Missing and redundant data points

So far we assumed that one data value is available for each of the model variables. For example, we assume that each metabolite level in each metabolic state has been measured, and that an *in-vitro* value is available for each kinetic constant. To account for the fact that values may be missing or measured multiple times, we need to relate a vector of model variables to the corresponding vector of variables measured (possibly, comprising multiple measurements of the same variable). The connection can be made by a data mapping matrix $\mathbf{D}_{\mathrm{x}}$ (for metabolite log concentrations, and similar mapping matrices for other variables) that maps the vector of metabolite log-concentrations in the model onto the vector of metabolite log-concentration data values. In the ideal case (all values have been measured exactly once), $\mathbf{D}_{\mathrm{x}}$ is a simple identity matrix. If a concentration has not been measured, the corresponding row of this identity matrix must be omitted, and if a value has been measured multiple times, the row must be duplicated. With mapping matrices, our likelihood loss score (with terms for kinetic constants and state variables) becomes

$$L^{\star}(\mathbf{x},\mathbf{z}) \quad = \quad \mathsf{quad}(\mathbf{D}_{\mathbf{q}}\,\mathbf{q}-\bar{\mathbf{q}}_{\mathrm{lk}},\boldsymbol{\Sigma}^{\mathbf{q}}_{\mathrm{lk}})+\mathsf{quad}(\mathbf{D}_{\mathrm{x}}\,\mathbf{x}-\bar{\mathbf{x}}_{\mathrm{lk}},\boldsymbol{\Sigma}^{x}_{\mathrm{lk}})+\mathsf{quad}(\mathbf{D}_{\mathrm{e}}\,\mathbf{z}-\bar{\mathbf{z}}_{\mathrm{lk}},\boldsymbol{\Sigma}^{z}_{\mathrm{lk}}), \tag{17}$$

and formula (6) for posterior means and covariances reads

$$\begin{aligned}
\boldsymbol{\Sigma}^{\star\mathbf{x}}_{\mathrm{po}} &= [\boldsymbol{\Sigma}^{x\;-1}_{\mathrm{pr}}+\mathbf{D}_{\mathrm{x}}^{\top}\boldsymbol{\Sigma}^{x\;-1}_{\mathrm{lk}}\mathbf{D}_{\mathrm{x}}]^{-1}\\
\bar{\mathbf{x}}^{\star}_{\mathrm{po}} &= \boldsymbol{\Sigma}^{\mathbf{x}\star}_{\mathrm{po}}\,[\boldsymbol{\Sigma}^{x\;-1}_{\mathrm{pr}}\,\bar{\mathbf{x}}_{\mathrm{pr}}+\mathbf{D}_{\mathrm{x}}^{\top}\,\boldsymbol{\Sigma}^{x\;-1}_{\mathrm{lk}}\,\bar{\mathbf{x}}_{\mathrm{lk}}].
\end{aligned} \tag{18}$$

Of course, missing and multiple values for kinetic constants, enzyme concentrations, and thermodynamic forces can be treated in the very same way (with analogous terms for $\mathbf{q}$, $\mathbf{z}$, and possibly $\boldsymbol{\theta}$). This is particularly important

for kinetic constants, where typically only a small fraction of the constants have been measured *in vitro*. Finally, instead of using mapping matrices, there is also a simpler practical solution. Whenever data values are missing, we can replace them by invented values with infinite error bars. Likewise, if multiple data values exist for one variable, we can merge them into a single value to be used as a data point. The calculation follows the same scheme a Eq. (18), with a vector $\bar{\mathbf{x}}_{\mathrm{lk}}$ containing the multiple measured data values, σ_{x_l} their standard deviations, mapping matrices $\mathbf{D} = (1, 1, ..)^{\top}$ relating a single model variable to multiple measured values, and no prior. We obtain

$$\begin{aligned} \sigma^2_{\mathrm{out}} &= [\mathbf{D}^{\top}\mathrm{diag}(\sigma^2_x)^{-1}\mathbf{D}]^{-1} = 1/\sum_l \sigma_{x_l}^{-2} \\ \bar{x}_{\mathrm{out}} &= \sigma^2_{\mathrm{out}}\,[\mathbf{D}\,\mathrm{diag}(\sigma^2_x)^{-1}\,\bar{\mathbf{x}}_{\mathrm{lk}}] = \frac{\sum_l \bar{x}_l/\sigma^2_x}{\sum_l 1/\sigma^2_{x_l}}. \end{aligned} \tag{19}$$

3 Parameter distributions

3.1 Parameter identifiability

Parameter identifiability To see what useful information can be extracted from data, we need to think about parameter identifiability and the usage of priors. In model fitting, parameters or parameter combinations may be non-identifiable: such parameters have no effect on measured variables, and therefore cannot be inferred from available data [9]. Similarly, if parameters have little effect on measured variables, and if data are just a bit noisy, our estimates of these parameters will be very uncertain. For example, if a reaction is strongly forward-driven, its backward k_{cat} and K_{M} values have little effect on state data and are hard to determine (compared to forward k_{cat} and K_{M} values); if an enzyme is always close to saturation, its K_{M} value is hard to infer, and if an enzyme is always in its linear range (because substrate levels are low), the $k_{\mathrm{cat}}/K_{\mathrm{M}}$ ratio may be well-determined, but not the k_{cat} and K_{M} individually; finally, enzyme concentrations and k_{cat} values appear in rate laws in the form of a product (which is therefore well-determined by the fluxes), but their ratios may be ill-determined. This means that a scaling an error in flux or enzyme data will have an immediate effect on k_{cat} estimates.

Problems arising from non-identifiability If model variables are non-identifiable (or poorly identifiable), this will cause large uncertainties in the posterior distribution. With a convex problem and non-flat priors, we know that the posterior mode is a single point in solution space. However, this point is somewhat arbitrary if the priors are broad, and posterior sampling would reveal that many other solutions (along the "non-identifiable subspace") are almost as good. This raises some practical questions. Can we improve the result by using more data (e.g., metabolite concentrations from more metabolic states)? How many metabolic states are needed to identify all kinetic parameters? Which variables are hard to infer, and which variables will remain non-identifiable, no matter how much state data are provided? Paradoxically, variables that are *always* non-identifiable (or poorly identifiable) are not a big problem, because these variables do not matter for model predictions. For example, if an enzyme is *always* close to saturation, we cannot determine its K_{M} value, but we also don't need it for simulation. Only if we simulate other conditions (in which the enzyme *is not* close to saturation), the results may be poor. Of course, identifiability issues are not specific to model balancing; other estimation methods would face the same problem.

3.2 Prior distributions

Importance and choice of priors Since non-identifiable parameters are estimated solely based on priors, the

choice of realistic priors is important! If data for variables are available, the usage of priors pushes the estimates towards the prior mean: high values will be estimated too low and low values will be estimated too high. This effect will be small if a variable has been measured precisely or if priors are very broad. However, for non-identifiable variables the effect is large: while model balancing predicts a single (posterior mode) value, this value is almost arbitrary, determined entirely by the choice of the prior, and carries a large uncertainty (marginal posterior variance). On the one hand, this calls for posterior sampling (instead of reporting only the posterior mode). On the other hand, this underlines the need for realistic, informative priors.

Informative priors To define informative priors for kinetic constants [10, 11], we followed the procedure from parameter balancing [1] which accounts for parameter dependencies. We start by assuming independent Gaussian priors for all independent constants and (independent Gaussian) pseudo values for dependent constants: taken together, they define a correlated prior for all constants. Non-diagonal covariance matrices for $\ln K_{\rm eq}$ values, obtained from equilibrator, could be taken into account. Mean values and standard deviations were taken from [12] and manually refined to match empirical distribution of kinetic constants. For the state variables (metabolite and enzyme log-concentrations), we typically use diagonal covariances $\mathbf{\Sigma}^{x}_{\rm lk} = {\rm diag}(\sigma^{\mathbf{x}}_{\rm lk})^2$ and $\mathbf{\Sigma}^{z}_{\rm lk} = {\rm diag}(\sigma^{\mathbf{z}}_{\rm lk})^2$, assuming independent measurement errors. However, correlated errors (e.g. caused by normalisation for metabolomics or proteomics samples) may be taken into account. There are several possible reasons to do this: first, concentrations differ more strongly between metabolites than between metabolic states for the same metabolite. This means, for the vector of all metabolite data, that values for each metabolite tend to be correlated. We could describe this by splitting the data values into $x_i^{(s)} = x_i^{\rm ref} + \Delta x_i^{(s)}$, with a reference value $x_i^{\rm ref}$ and deviations $\Delta x_i^{(s)}$. Assuming uncorrelated priors for $x_i^{\rm ref}$ and $\Delta x_i^{(s)}$ entails, directly, a correlated prior for $x_i^{(s)}$ (and the same for enzyme log concentrations z_l). Second, if we model metabolic time series, and assume that state variables vary smoothly over time, we can implement this assumption by imposing a prior that assumes correlations between (one and the same concentration at) subsequent time points. Third, there may even be reasons to assume correlated errors in data values: for example, in metabolomics data, differences in sample preparation or a normalisation per sample will lead to correlated measurement errors (by which, for example, all metabolite concentrations in one sample may appear too high or too low). In theory, such effects could be accounted for by assuming a correlated covariance matrix in the likelihood terms.

Choice of priors and constraints To define priors in practice, pseudo values, and constraints (for kinetic constants, metabolite concentrations and enzyme concentrations), we used the default values from parameter balancing (see www.parameterbalancing.net). However, when running parameter balancing as a test, we found that the available $k_{\rm cat}$ values were typically much higher than the prior median value as expected for enzymes in central metabolism [13]. In view of these data, we changed the prior for $k_{\rm cat}$ values from a median of 10 s^{-1} (geometric standard deviation 100) to a median of 200 s^{-1} (geometric standard deviation 50). Likewise, we changed the prior width for $K_{\rm M}$ values from a geometric standard deviation of 10 to a geometric standard deviation of 20 (while keeping the median 0.1 mM unchanged). A table describing the priors is provided in the github repository, file resources/data/data-prior/model-balancing_prior.tsv. These values, used in the matlab implementation, can easily be modified. Importantly, in the actual model balancing calculations, a much broader prior was used in order to put more weight on existing data.

3.3 Artificial data

Artificial data were generated as follows:

- **Artificial kinetic constants** Artificial data for kinetic constants are generated as follows. Given a network structure, true artificial kinetic constants are generated by assigning random (log-normal) values to the basic kinetic constants ($\ln K_{\rm eq}^{\rm ind}$, $\ln K_M$, and $\ln K_V$), using the same distributions that are also used as priors in

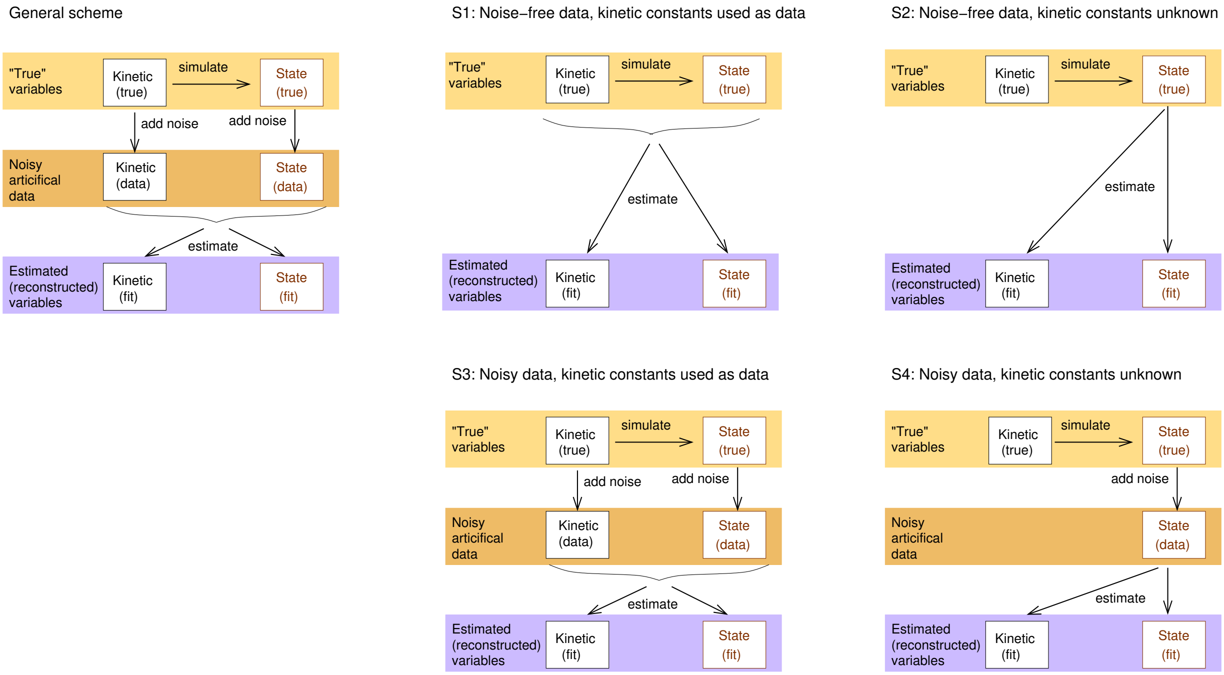

Figure 2: Model balancing: estimation scenarios with artificial data. Left: general procedure. Kinetic constants for a given model are drawn from random distributions (respecting their dependencies), and metabolic state data are generated by combining sampling and simulation runs (top row). From these "true" values, artificial kinetic and state data are generated by adding uncorrelated noise (centre row). Kinetic parameters and metabolic state variables (bottom row) are estimated by model balancing and compared to the true values. Right: Four variants of this procedure (called S1-S4): with or without noise, and with or without usage of kinetic constants as data (or as a third option, with usage of equilibrium constants only).

model balancing. Derived kinetic constants are computed from the basic constants, and Gaussian random variables are added on logarithmic scale. In the data structures, the "true" artificial data (without noise) are given in `true`, while artificial data with noise (or no noise, depending on the scenario) are given in `kinetic_data`. The artificial data contain no estimates of the error bars; for these error bars, information from the prior table is used.

- **Artificial metabolic state data: small test models** To generate artificial data for metabolic states, enzyme concentrations and external metabolite concentrations are randomly sampled from the same distributions that are used as priors in model balancing. Then the model is parameterised with the "true" artificial kinetic constants and solved to obtain a steady reference state (steady-state metabolite concentrations and fluxes). Based on this reference state, a number of metabolic states are constructed by randomly varying metabolite and enzyme concentrations (again, following the prior distribution) and computing the (non-steady) reaction rates[6]. The resulting states are seen as "true values". To generate noisy state data, uncorrelated random noise is added.

- **Artificial metabolic state data: *E. coli* model** Artificial data for the *E. coli* model, generated as described, turned out to be very unrealistic: the fluxes did not show any resemblance at all to metabolic fluxes observed in central metabolism. For more realistic test scenarios, we generates artificial datat for this model, based known metabolic states. We first initialised fluxes and metabolite concentrations with realistic values from ECM [3], and then multiplied the enzyme levels by log-normal random variables with a geometric mean of 1 and a geometric standard deviation of 1.5. The equilibrium constants were taken from ECM. Fructose bisphosphatase (which is inactive in this state) was shut down to 10^{-8} to obtain plausible states.

[6] Alteratively, state data could also be snapshots from simulated dynamic time course (fluxes, metabolite concentrations and enzyme concentrations).

- **Flux directions** Artificial flux data contain additive noise. However, since infeasible flux directions are not allowed, noise is omitted whenever the flux direction would change.

4 Enzyme demand: a formula for disciplined convex programming

Disciplined Convex Programming [4] is a framework for convex optimisation in which optimality problems are guaranteed to be convex. To this end, objectives and constraints must be defined by formulae composed of defined sets of mathematical functions. Here we show that a central formula in model balancing, the formula for the logarithmic enzyme demand as a function of logarithmic kinetic constants and metabolite concentrations, can be expressed in this way.

We consider a reaction with stoichiometric coefficients in a sparse vector $\mathbf{n}$ and K_{M} values in a sparse vector $\mathbf{k}$. Metabolite concentrations are given in a vector $\mathbf{c}$. All three vectors are columns of length n_{met} (number of metabolites in the network). For convenience we define the molecularities $m^{\mathrm{s}}_{\alpha} = |n_{\alpha}|$ if $n_{\alpha} < 0$ (0 otherwise) for substrates and $m^{\mathrm{p}}_{\beta} = |n_{\beta}|$ if $n_{\beta} > 0$ (0 otherwise) for products. The denominator of the common modular rate law [14] reads

$$D_{\mathrm{cm}} = \prod_{\alpha}(1 + c_{\alpha}/k_{\alpha})^{m^{\mathrm{s}}_{\alpha}} + \prod_{\beta}(1 + c_{\beta}/k_{\beta})^{m^{\mathrm{p}}_{\beta}} - 1. \tag{20}$$

Effectively, the first product contains only terms for substrates (all other terms are equal to 1 and can be neglected). An exponent $m^{\mathrm{s}}_i = 2$ means, effectively, that the same term appears twice in the product (analogous, three times for $m^{\mathrm{s}}_i = 3$, and so on). So we can do the counting differently: instead of iterating over metabolites, we iterative over the list of substrates, where substrates can be listed several times depending on their molecularity. For each index i in this list, we define the metabolite index (pointing to elements in the vector $\mathbf{c}$) by $\alpha(i)$. In case of molecularities larger than 1, several i map to the same α. With similar definitions for products, we obtain

$$D_{\mathrm{cm}} = \prod_{i}(1 + c_{\alpha(i)}/k_{\alpha(i)}) + \prod_{l}(1 + c_{\beta(l)}/k_{\beta(l)}) - 1. \tag{21}$$

We now use the abbreviation $y^{\mathrm{s}}_i = \ln(\frac{c_{\alpha(i)}}{k_{\alpha(i)}})$. The vector $\mathbf{y}^{\mathrm{s}}$ can be written, for convenience, as

$$\mathbf{y}^{\mathrm{s}} = \mathbf{T}^{\mathrm{s}}\,(\ln(\mathbf{c}) - \ln(\mathbf{k})) = \mathbf{T}^{\mathrm{s}}\,\ln(\mathbf{c}) - \mathbf{T}^{\mathrm{s}}\,\ln(\mathbf{k}) \tag{22}$$

with a matrix $\mathbf{T}^{\mathrm{s}}$ (of size = length of substrate list $\times n_{\mathrm{met}}$) that contains, in each row i, a single entry =1 in column $\alpha(i)$. For the product side, we obtain a similar vector $\mathbf{y}^{\mathrm{p}}$ and a matrix $\mathbf{T}^{\mathrm{p}}$. Now our denominator reads, in short,

$$D_{\mathrm{cm}} = \prod_{i}(1 + \mathrm{e}^{y^{\mathrm{s}}_i}) + \prod_{l}(1 + \mathrm{e}^{y^{\mathrm{p}}_l}) - 1, \tag{23}$$

and by reordering the terms we obtain

$$\begin{aligned} D_{\mathrm{cm}} &= 1 \quad + \sum_{i} \mathrm{e}^{y^{\mathrm{s}}_i} + \sum_{i \neq j} \mathrm{e}^{y^{\mathrm{s}}_i}\mathrm{e}^{y^{\mathrm{s}}_j} + \sum_{i \neq j \neq k} \mathrm{e}^{y^{\mathrm{s}}_i}\mathrm{e}^{y^{\mathrm{s}}_j}\mathrm{e}^{y^{\mathrm{s}}_k} + ... \\ &\qquad + \sum_{l} \mathrm{e}^{y^{\mathrm{p}}_l} + \sum_{l \neq q} \mathrm{e}^{y^{\mathrm{p}}_l}\mathrm{e}^{y^{\mathrm{p}}_q} + \sum_{l \neq q \neq r} \mathrm{e}^{y^{\mathrm{p}}_l}\mathrm{e}^{y^{\mathrm{p}}_q}\mathrm{e}^{y^{\mathrm{p}}_r} + ... \end{aligned} \tag{24}$$

with indices $i, j, k, ..$ from the substrate list and indices $l, q, r, ..$ from the product list. The maximal size of tuples, for the substrate side, is given by the length of the substrate list (and accordingly for the product side). We next

write this as

$$\begin{aligned}D_{\mathrm{cm}} &= 1 \quad + \sum_i \mathrm{e}^{y_i^{\mathrm{s}}} + \sum_{i \neq j} \mathrm{e}^{y_i^{\mathrm{s}} + y_j^{\mathrm{s}}} + \sum_{i \neq j \neq k} \mathrm{e}^{y_i^{\mathrm{s}} + y_j^{\mathrm{s}} + y_k^{\mathrm{s}}} + \ldots \\ & \qquad + \sum_l \mathrm{e}^{y_l^{\mathrm{p}}} + \sum_{l \neq q} \mathrm{e}^{y_l^{\mathrm{p}} + y_q^{\mathrm{p}}} + \sum_{l \neq q \neq r} \mathrm{e}^{y_l^{\mathrm{p}} + y_q^{\mathrm{p}} + y_r^{\mathrm{p}}} + \ldots \end{aligned} \tag{25}$$

Now, for the sake of example, we assume a reaction with four substrate molecules (substrate list of length 4). We define matrices with rows corresponding to all single items, pairs, triples, and quadruples (and columns corresponding to the elements of $\mathbf{y}^{\mathrm{s}}$):

$$\begin{aligned}\mathbf{K}_1^{(4)} &= \begin{pmatrix} 1 & 0 & 0 & 0 \\ 0 & 1 & 0 & 0 \\ 0 & 0 & 1 & 0 \\ 0 & 0 & 0 & 1 \end{pmatrix}, \quad \mathbf{K}_2^{(4)} = \begin{pmatrix} 1 & 1 & 0 & 0 \\ 1 & 0 & 1 & 0 \\ 1 & 0 & 0 & 1 \\ 0 & 1 & 1 & 0 \\ 0 & 1 & 0 & 1 \\ 0 & 0 & 1 & 1 \end{pmatrix} \\ \mathbf{K}_3^{(4)} &= \begin{pmatrix} 1 & 1 & 1 & 0 \\ 1 & 1 & 0 & 1 \\ 1 & 0 & 1 & 1 \\ 0 & 1 & 1 & 1 \end{pmatrix}, \quad \mathbf{K}_4^{(4)} = \begin{pmatrix} 1 & 1 & 1 & 1 \end{pmatrix} \end{aligned} \tag{26}$$

and combine them in a matrix

$$\mathbf{K}^{(4)} = \begin{pmatrix} \mathbf{K}_1^{(4)} \\ \mathbf{K}_2^{(4)} \\ \mathbf{K}_3^{(4)} \\ \mathbf{K}_4^{(4)} \end{pmatrix}. \tag{27}$$

With this matrix, the single sum terms from the first line, written as a vector, are given by $\mathrm{e}^{\mathbf{K}^{(4)}\,\mathbf{y}^{\mathrm{s}}}$, where the exponential function is applied componentwise to the vectorial exponent. If we generalise this from the case $n_{\mathrm{s}} = 4$ to substrate lists of any length, we obtain the respective matrix $\mathbf{K}^{(n_{\mathrm{s}})}$. Altogether, for a reaction with substrate list length n_{s} and product list length n_{p}, the exponent terms in D_{cm} are given by the vector

$$\mathbf{z} = \begin{pmatrix} 0 \\ \mathbf{K}^{n_{\mathrm{s}}}\,\mathbf{y}^{\mathrm{s}} \\ \mathbf{K}^{n_{\mathrm{p}}}\,\mathbf{y}^{\mathrm{p}} \end{pmatrix}. \tag{28}$$

D_{cm} itself is given by $D_{\mathrm{cm}} = \sum_q \mathrm{e}^{z_q}$. As mentioned before, $\mathbf{y}^{\mathrm{s}}$ and $\mathbf{y}^{\mathrm{p}}$ can be written as $\mathbf{y}^{\pm} = \mathbf{A}^{\pm} \ln \mathbf{c} + \mathbf{b}^{\pm}$, so the overall formula becomes

$$D_{\mathrm{cm}} = \sum_l \mathrm{e}^{\mathbf{a}_l (\ln \mathbf{c} - \ln \mathbf{k})}. \tag{29}$$

The vectors $\mathbf{a}_l$ are the rows of the matrix

$$\mathbf{A} = \begin{pmatrix} \mathbf{0} \\ \mathbf{K}^{n_{\mathrm{s}}}\,\mathbf{T}^{\mathrm{s}} \\ \mathbf{K}^{n_{\mathrm{p}}}\,\mathbf{T}^{\mathrm{p}} \end{pmatrix}, \tag{30}$$

where $\mathbf{0}$ is a zero row vector. Now our aim is to obtain a convex expression for $1/\eta^{\mathrm{kin}} = D_{\mathrm{cm}}/D_{\mathrm{sm}}$ with

$D_{\rm sm} = \prod_i \frac{c_{\alpha(i)}}{k_{\alpha(i)}}$. By writing $D_{\rm sm}$ as

$$D_{\rm sm} = {\rm e}^{\sum_i y_i^{\rm s}} = {\rm e}^{\mathbf{1} S^{\rm s} (\ln \mathbf{c} - \ln \mathbf{k})}, \tag{31}$$

we obtain

$$\frac{1}{\eta_{\rm cm}^{\rm kin}} = \frac{D_{\rm cm}}{D_{\rm sm}} = \sum_l {\rm e}^{\mathbf{a}_l (\ln \mathbf{c} - \ln \mathbf{k}) - \mathbf{1} S^{\rm s} (\ln \mathbf{c} - \ln \mathbf{k})} = \sum_l {\rm e}^{\mathbf{b}_l (\ln \mathbf{c} - \ln \mathbf{k})}, \tag{32}$$

where vectors $\mathbf{b}_l$ are the rows of the matrix

$$\mathbf{B} = \mathbf{A} - (\mathbf{1}\,\mathbf{1}^\top)\,\mathbf{T}^{\rm s}. \tag{33}$$

Finally, the term $-\ln \eta_{\rm cm}^{\rm kin}$ can be written as the log of sum of exponentials of the vector $\mathbf{B}(\ln \mathbf{c} - \ln \mathbf{k})$. We obtain

$$\ln \eta_{\rm cm}^{\rm kin} = -\ln \frac{D_{\rm cm}}{D_{\rm sm}} = -\text{LogSumExp}\left(\left[\begin{pmatrix} \mathbf{0} \\ \mathbf{K}^{n_{\rm s}}\, \mathbf{T}^{\rm s} \\ \mathbf{K}^{n_{\rm p}}\, \mathbf{T}^{\rm p} \end{pmatrix} - (\mathbf{1}\,\mathbf{1}^\top) S^{\rm s} \right] (\ln \mathbf{c} - \ln \mathbf{k}) \right), \tag{34}$$

a formula compliant with disciplined convex programming.

References

[1] T. Lubitz, M. Schulz, E. Klipp, and W. Liebermeister. Parameter balancing for kinetic models of cell metabolism. *J. Phys. Chem. B*, 114(49):16298–16303, 2010.

[2] E. Noor, A. Flamholz, W. Liebermeister, A. Bar-Even, and R. Milo. A note on the kinetics of enzyme action: a decomposition that highlights thermodynamic effects. *FEBS Letters*, 587(17):2772–2777, 2013.

[3] E. Noor, A. Flamholz, A. Bar-Even, D. Davidi, R. Milo, and W. Liebermeister. The protein cost of metabolic fluxes: prediction from enzymatic rate laws and cost minimization. *PLoS Computational Biology*, 12(10):e1005167, 2016.

[4] M. Grant, S. Boyd, and Y. Ye. chapter Disciplined Convex Programming. Springer, 2006.

[5] Model balancing documentation on https://model-balancing.readthedocs.io. Accessed: 2021-09-15.

[6] T.B. Jacobson, P.A. Adamczyk, D.M. Stevenson, M. Regner, J. Ralph, J.L. Reed, and D. Amador-Noguez. 2h and 13c metabolic flux analysis elucidates in vivo thermodynamics of the ed pathway in zymomonas mobilis. *Metabolic Engineering*, 54:301–316, 2019.

[7] T. Lubitz, J. Hahn, F.T. Bergmann, E. Noor, E. Klipp, and W. Liebermeister. SBtab: A flexible table format for data exchange in systems biology. *Bioinformatics*, 32(16):2559–2561, 2016.

[8] M. Hucka, A. Finney, H.M. Sauro, H. Bolouri, J.C. Doyle, H. Kitano, A.P. Arkin, B.J. Bornstein, D. Bray, A. Cornish-Bowden, A.A. Cuellar, S. Dronov, E.D. Gilles, M. Ginkel, V. Gor, I.I. Goryanin, W.J. Hedley, T.J. Hodgman, J.H. Hofmeyr, P.J. Hunter, N.S. Juty, J.L. Kasberger, A. Kremling, U. Kummer, N. Le Novère, L.M. Loew, D. Lucio, P. Mendes, E. Minch, E.D. Mjolsness, Y. Nakayama, M.R. Nelson, P.F. Nielsen, T. Sakurada T J.C. Schaff, B.E. Shapiro, T.S. Shimizu, H.D. Spence, J. Stelling, K. Takahashi, M. Tomita, J. Wagner, J. Wang, and the SBML Forum. The Systems Biology Markup Language (SBML): A medium for representation and exchange of biochemical network models. *Bioinformatics*, 19(4):524–531, 2003.

[9] S. Srinivasan, W.R. Cluett, and R. Mahadevan. A scalable method for parameter identification in kinetic models of metabolism using steady state data. *Bioinformatics*, page btz445, 2019.

[10] W. Liebermeister and E. Klipp. Bringing metabolic networks to life: integration of kinetic, metabolic, and proteomic data. *Theor. Biol. Med. Mod.*, 3:42, 2006.

[11] A. Tsigkinopoulou, A. Hawari, M. Uttley, and R. Breitling. Defining informative priors for ensemble modeling in systems biology. *Nature Protocols*, 13(11):2643, 2018.

[12] T. Lubitz and W. Liebermeister. Parameter balancing: consistent parameter sets for kinetic metabolic models. *Bioinformatics*, 35:3857–3858, 2019.

[13] A. Bar-Even, E. Noor, Y. Savir, W. Liebermeister, D. Davidi, D.S. Tawfik, and R. Milo. The moderately efficient enzyme: evolutionary and physicochemical trends shaping enzyme parameters. *Biochemistry*, 21:4402–4410, 2011.

[14] W. Liebermeister, J. Uhlendorf, and E. Klipp. Modular rate laws for enzymatic reactions: thermodynamics, elasticities, and implementation. *Bioinformatics*, 26(12):1528–1534, 2010.
